# San-Huang-Yi-Shen Capsule Ameliorates Diabetic Nephropathy in Rats Through Modulating the Gut Microbiota and Overall Metabolism

**DOI:** 10.3389/fphar.2021.808867

**Published:** 2022-01-04

**Authors:** Xiuhai Su, Wenxia Yu, Airu Liu, Congxiang Wang, Xiuzhen Li, Juanjuan Gao, Xiaofei Liu, Wenhui Jiang, Yue Yang, Shuquan Lv

**Affiliations:** Cangzhou Hospital of Integrated TCM and Western Medicine of Hebei Province, Cangzhou, China

**Keywords:** San-Huang-Yi-Shen capsule, diabetic nephropathy, gut microbiota, arginine biosynthesis, tricarboxylic acid cycle, tyrosine metabolism, arginine and proline metabolism

## Abstract

San-Huang-Yi-Shen capsule (SHYS) has been used in the treatment of diabetic nephropathy (DN) in clinic. However, the mechanisms of SHYS on DN remain unknown. In this study, we used a high-fat diet (HFD) combined with streptozotocin (STZ) injection to establish a DN rat model. Next, we used 16S rRNA sequencing and untargeted metabolomics to study the potential mechanisms of SHYS on DN. Our results showed that SHYS treatment alleviated the body weight loss, hyperglycemia, proteinuria, pathological changes in kidney in DN rats. SHYS could also inhibite the oxidative stress and inflammatory response in kidney. 16S rRNA sequencing analysis showed that SHYS affected the beta diversity of gut microbiota community in DN model rats. SHYX could also decrease the *Firmicutes* to *Bacteroidetes* (*F* to *B*) ratio in phylum level. In genus level, SHYX treatment affected the relative abundances of *Lactobacillus*, *Ruminococcaceae UCG-005*, *Allobaculum*, *Anaerovibrio*, *Bacteroides* and *Candidatus_Saccharimonas.* Untargeted metabolomics analysis showed that SHYX treatment altered the serum metabolic profile in DN model rats through affecting the levels of guanidineacetic acid, L-kynurenine, prostaglandin F1α, threonine, creatine, acetylcholine and other 21 kind of metabolites. These metabolites are mainly involved in glycerophospholipid metabolism, tryptophan metabolism, alanine, aspartate and glutamate metabolism, arginine biosynthesis, tricarboxylic acid (TCA) cycle, tyrosine metabolism, arginine and proline metabolism, arginine and proline metabolism, phenylalanine, tyrosine and tryptophan biosynthesis, phenylalanine metabolism, and D-glutamine and D-glutamate metabolism pathways. Spearman correlation analysis showed that *Lactobacillus, Candidatus_Saccharimonas, Ruminococcaceae UCG-005, Anaerovibrio, Bacteroides,* and *Christensenellaceae_R-7_group* were closely correlated with most of physiological data and the differential metabolites following SHYS treatment. In conclusion, our study revealed multiple ameliorative effects of SHYS on DN including the alleviation of hyperglycemia and the improvement of renal function, pathological changes in kidney, oxidative stress, and the inflammatory response. The mechanism of SHYS on DN may be related to the improvement of gut microbiota which regulates arginine biosynthesis, TCA cycle, tyrosine metabolism, and arginine and proline metabolism.

## 1 Introduction

Diabetic nephropathy (DN), major complications of diabetes, is a contributing factor in late-stage renal failure (Wang et al., 2016). The current treatment for DN includes blood glucose control and regulation of lipid metabolism using anti-hypertension medicines (Mann et al., 2010; Pazdro and Burgess, 2010; Tone et al., 2005; Jin et al., 2014). However, these conventional treatments do not completely prevent the progression of DN (Tuttle et al., 2018). The development of new therapeutic agents to improve renal function and halt the progression of DN has become an active area of investigation.

Traditional Chinese medicine (TCM) has contributed significantly to the treatment of DN. Numerous studies have shown that TCM has significant advantages for improving renal function, controlling blood glucose levels, and inhibiting the inflammatory response on DN (Zhong et al., 2015). A multicenter randomized controlled study showed that Liu-Wei-Di-Huang pill significantly reduces erythrocyte aldose reductase activity, β2-microglobulin expression, and urinary protein levels in diabetic patients (Song et al., 2004). Qi-Zhi-Jiang-Tang decoction also delays the progression of DN (Guo et al., 2014). Wu-Ling powder ameliorates the inflammatory response in a rat model of DN by inhibiting the NF-κB signaling pathway (Liu et al., 2009). Tong-Shen-Luo (TSL) significantly reduces the extracellular matrix in renal tissues in a rat model of DN (Wu et al., 2007). Finally, Xian-Zhen decoction decreases the accumulation of glycosylated products in renal tissues of rats with DN (Tang et al., 2005).

The human intestine contains a plethora of microorganisms that interact with each other and together maintain the metabolic-immune nervous system homeostasis of the body (Li et al., 2020). Disorders in gut microbiota contribute to the development of various diseases (Guarner, 2006). Patients with DN have an increased ratio of *Firmicutes* to *Bacteroides* in the gut compared with healthy individuals (Gong et al., 2021). These alterations in the microbiota are associated with various pathological changes associated with DN, abnormal hemodynamic inflammatory responses, and metabolic abnormalities (Grigor’eva, 2020; Magne et al., 2020; Macho-González et al., 2021; Zhang et al., 2021). Transplantation of germ-free mice with microbiota from mice with DN resulted in elevated levels of 24-h urine proteins (Cai et al., 2020). Furthermore, gut microbiota may be involved in the progression of DN by modulating metabolism (Li et al., 2020). The pathogenesis of DN may be elucidated by analyzing microbiota metabolism using 16S rRNA sequencing technology combined with untargeted metabolomics, This may lead to the development of new treatments for DN that regulate gut microbiota and host metabolism.

San-Huang-Yi-Shen capsule (SHYS) consists of *Astragalus mongholicus* Bunge, *Panax quinquefolius* L., *Dioscorea oppositifolia* L., *Cornus officinalis* Siebold and Zucc., *Cuscuta chinensis* Lam., *Polygonatum sibiricum* Redouté, *Rehmannia glutinosa* (Gaertn.) DC., *Euryale ferox* Salisb., *Rosa laevigata* Michx., *Leonurus japonicus* Houtt., *Salvia miltiorrhiza* Bunge, *Conioselinum anthriscoides* ‘Chuanxiong’, *Atractylodes lancea* (Thunb.) DC., *Paeonia lactiflora* Pall., and *Gypsophila vaccaria* (L.) Sm. And has been used in the treatment of many chronic renal diseases including DN, IgA nephropathy and chronic renal failure (Bian et al., 2011; Su et al., 2015b; Zhong et al., 2016). Clinical studies showed that SHYS could alleviate the proteinuria in DN patients (Su et al., 2015a). Animal study has also demonstrated the protective effects of SHYS on DN model rats (Chi et al., 2013). However, the mechanisms of SHYS on DN remain unknown. In this study, we used a high-fat diet (HFD) combined with streptozotocin (STZ) injection to establish a DN rat model. Next, we used 16S rRNA sequencing and untargeted metabolomics to study the potential mechanisms of SHYS on DN.

## 2 Materials and Methods

### 2.1 Reagents

HFD (65.75% basal chow, 20% sucrose, 10% lard, 3% egg yolk powder, 1% cholesterol, 0.25% pig bile salt) was purchased from Beijing Sibeifu Bioscience Co., Ltd. (Beijing, China). Irbesartan was obtained from Sanofi Winthrop Industrie (France). STZ was purchased from Solarbio Biotechnology Co., Ltd. (Beijing, China). Creatinine (Cr), blood urea nitrogen (BUN), urine protein, superoxide dismutase (SOD), methane dicarboxylic aldehyde (MDA), and glutathione peroxidase (GSH-Px) assay kits test kits were obtained from Nanjing Jiancheng Biological Engineering Institute (Nanjing, China). Rat interleukin (IL)-1β, IL-6, tumor necrosis factor alpha (TNF-α) enzyme-linked immunosorbent assay (ELISA) kit was obtained from Multi Science Biotechnology Co., Ltd. (Hangzhou, China). Reference standards of ferulic acid, atractylodin, tanshinone, astragaloside, hyperoside, loganin, gallic acid, morroniside, allantoin, oleanic acid, ginsenoside Rb1, ginsenoside Re, ginsenoside Rg1, paeoniflorin, vaccarin, pyrrolidinium, leonurine, and catalpol were obtained from Sichuan Weikeqi Biological Technology Co., Ltd. (Sichuan, China).

### 2.2 Quality Control and Analysis of Main Compounds in SHYS

SHYS was prepared from the pharmacy department of Cangzhou Hospital of Integrated Traditional Chinese and Western Medicine. Briefly, 15 g of *Astragalus mongholicus* Bunge (Batch number: 201126), 12 g of *Panax quinquefolius* L., 12 g of *Dioscorea oppositifolia* L. (Batch number: 201008), 12 g of *Cornus officinalis* Siebold and Zucc. (Batch number: 201126), 12 g of *Cuscuta chinensis* Lam. (Batch number: 200815), 12 g of *Polygonatum sibiricum* Redouté (Batch number: 200912), 15 g of *Rehmannia glutinosa* (Gaertn.) DC. (Batch number: 201126), 12 g of *Euryale ferox* Salisb. (Batch number: 201012), 12 g of *Rosa laevigata* Michx. (Batch number: 201126), 10 g of *Leonurus japonicus* Houtt. (Batch number: 200817), 12 g of *Salvia miltiorrhiza* Bunge (Batch number: 201102), 12 g of *Conioselinum anthriscoides* ‘Chuanxiong’ (Batch number: 200817), 10 g of *Atractylodes lancea* (Thunb.) DC. (Batch number: 201014), 10 g of *Paeonia lactiflora* Pall. (Batch number: 201012), and 6 g of *Gypsophila vaccaria* (L.) Sm. (Batch number: 201106) were weighed. All herbs were authenticated by pharmacist in the pharmacy department of Cangzhou Hospital of Integrated Traditional Chinese and Western Medicine. Then, these crude herbs were decocted, evaporated and made into capsuled prepapation according to the medical institution preparation standard in Hebei (approval number: Z20050795). The specification of SHYS was 0.45 g per capsule. The production licence of SHYS was shown in Supplementary Figure S1.

Quality control of SHYS was performed using Ultra performance liquid chromatography (UPLC; ACQUITY UPLC^®^, United States) coupled with Xevo G2 quadrupole-time of-flight (Q-TOF) mass spectrometer (MS; Waters Corp., Milford, MA, United States) systems. Briefly, the test solution was injected onto an ACQUITY UPLC BEH C18 Column (2.1 mm × 100 mm, 1.7 μm) held at 50°C. The flow rate was 0.3 ml/min and the injection volumn was 2 μl. Mobile phase A was 0.1% formic acid aqueous solution and mobile phase B was acetonitrile contained 0.1% formic acid. The mobile phase conditions were: 0 min, 5% B; 1 min, 10% B; 6 min 60% B; 6.5 min 100% B; 10 min 100% B; 10.1 min 5% B; 13 min 5% B.

A Q-TOF MS equipped with an electrospray ionization (ESI) source was used for both positive and negative ionization scan modes (m/z ranges from 50 to 1,200 Da). The scan time was 0.2 s. The capillary voltages were 3,000 V (positive mode) and 2,200 V (negative mode), respectively. The desolvation temperature was 350°C and the source temperature was 100°C. The sample cone voltage was 40 V and the extraction cone voltage was 4 V. The cone gas flow was 40 L/h and the desolvation gas flow was 800 L/h (both positive and negative modes).

### 2.3 Animals

Sixty male Sprague-Dawley (SD) rats (speciic pathogen free grade, 6–8 weeks old) weighing 200 ± 20 g, were obtained from the Beijing Huafukang Biotechnology Co., Ltd. [Production License No.: SCXK (Beijing) 2020-0016]. The animal were housed at 25 ± 2°C, a 12 h light-dark cycle, with free access to food and water. The study was approved by the Ethics Committee of Cangzhou Hospital of Integrated Traditional Chinese and Western Medicine.

### 2.4 Induction of DN Rat Model

DN model was induced using HFD and STZ injection as described previously (Shen et al., 2020). Briefly, rats were fed with HFD for 8 weeks. Subsequently, rats were injected intraperitoneally with 30 mg/kg of STZ (dissolved in 0.1 mol/L citric acid buffer, pH = 4.5). After STZ injection, rats were continued to be fed with HFD and blood glucose and 24-h urine protein levels after fasted for 12 h were investigated weekly. FBG levels of ≥12 mmol/L and urine protein levels of ≥20 mg/24 h were used as criteria for establishing the animal model of DN (Zhang et al., 2014), this occurred 2 weeks after STZ injection.

### 2.5 Animal Grouping

**FIGURE 1:**
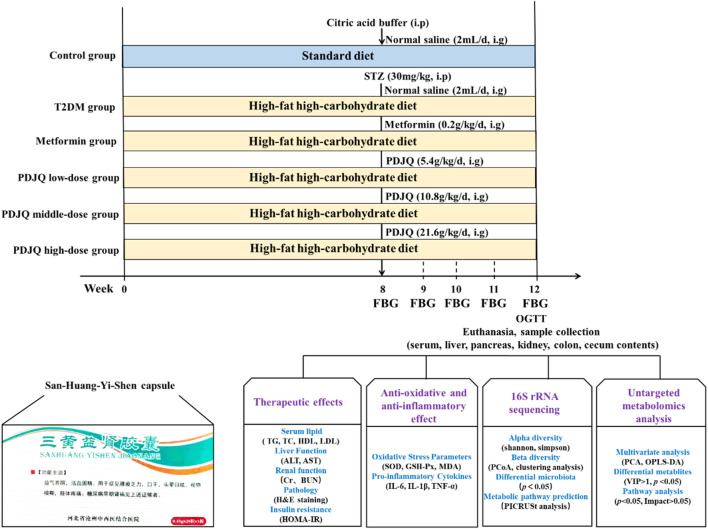
Overview of the experimental design for all groups.

After 1 week of acclimatization, 10 rats were randomly selected as the Control group and provided a standard diet, whereas the remaining 50 rats received HFD and STZ injection to induce DN. After the animal model had been established, DN rats were randomly divided into the Model, Irbesartan, SHYS low-dose, SHYS middle-dose, and SHYS high-dose groups, with 10 rats in each group. Rats in Control and Model groups were gavaged with 2 ml of distilled water; Rats in Irbesartan were gavaged with 11.51 mg/kg irbesartan (Wang et al., 2018); while rats in SHYS low-dose, SHYS middle-dose and SHYS high-dose groups were gavaged with SHYS capsule doses of 0.41, 0.81, and 1.62 g/kg, respectively, once daily for 4 consecutive weeks. The dosage in SHYS middle-dose group in our study was based on a conversion of human to rat body surface area, while the low dose was half the middle dose and high dose was double the middle dose. Body weight and FBG were measured every 2 weeks after SHYS treatment (Figure 1).

### 2.6 Measurement of Biochemical Indicators

After 4 weeks of SHYS intervention, 24 h urine samples were collected from all rats using metabolic cages. Then, rats were anesthetized with an intraperitoneal injection of 50 mg/kg pentobarbital sodium. Blood was collected from the inner canthus, and centrifuged at 3,000 rpm for 15 min to collect the serum. The levels of 24-h urine protein and serum creatine (Cr) and blood urea nitrogen (BUN) in each group of rats were determined using the test kits according to the manufacturer’s instructions (Nanjing Jiancheng Biological Engineering Institute).

The rats were sacrificed by cervical dislocation after blood sampling. The kidneys were quickly removed. 0.1 g of kidney tissues were weighed and immersed in 900 μl of normal saline and homogenized at 4°C. The supernatants were collected after centrifugation at 3,000 rpm for 15 min to obtain the renal tissue homogenate. Level of the total protein content in renal tissue homogenate was detected by BCA assay according to the manufacturer’s instructions (Nanjing Jiancheng Biological Engineering Institute). Besides, SOD and GSH-Px activities and MDA levels representing oxidative stress were measured according to the instructions described in the kit (Nanjing Jiancheng Biological Engineering Institute).

### 2.7 ELISA

After 4 weeks of SHYS intervention, IL-6, IL-1β, and TNF-α levels in renal tissue homogenates from each group were measured by ELISA, which was performed based on the manufacturer’s instructions (Multi Science Biotechnology Co., Ltd.).

### 2.8 Histopathology Staining of Renal Tissues

After 4 weeks of SHYS intervention, the renal tissues from each group were fixed in 10% formalin, washed with water for 20 min, dehydrated in gradient alcohol, cleared with xylene, embedded in paraffin. The tissues were cut into 5 μm thick strips, stained with hematoxylin and eosin (H and E), and subjected to Masson and Sirius Red staining. The relative collagenous fiber area was quantified using integrated optical density (IOD) with the Image-Pro Plus 6.0 software. The positive area (%) was calculated according to the following formula: IOD/sum area × 100%.

### 2.9 16S rRNA Sequencing

#### 2.9.1 Extraction of Fecal Genomic DNA

After 4 weeks of SHYS intervention, cecum contents were collected and weighed. Total genomic DNA was extracted using the CTAB/SDS method. The purity and concentration of DNA samples were assessed on a 1% agarose gel. The DNA was diluted to 1 ng/μl with sterile water.

#### 2.9.2 Polymerase Chain Reaction Amplification and Sequencing of 16S rRNA Gene

The V3-V4 region of the 16S rRNA gene was amplified using 338 F (5'-ACT​CCT​ACG​GGA​GGC​AGC​AG-3') and 806R (5'-GGACTACHVGGGTWTCTAAT-3') primers. PCR amplification included 10 ng of template DNA, 0.2 µm of forward and reverse primers, and 15 µl of Phusion^®^ High-Fidelity PCR Master Mix (New England Biolabs). The reaction conditions were as follows: pre-denaturation at 98°C for 1 min, 15 cycles of denaturation at 95°C for 10 s, annealing at 50°C for 30 s and extension at 72°C for 30 s, and then holding at 72°C for 5 min and storage at 4°C. The PCR products were purified using the Qiagen Gel Extraction Kit (Qiagen, Germany). A 2% agarose gel was used for detection. Sequencing libraries were generated using the TruSeq^®^ DNA PCR-Free Sample Preparation Kit (Illumina, United States) and library quality was assessed on a Qubit@ 2.0 Fluorometer (Thermo Scientific) and an Agilent Bioanalyzer 5400 system (Agilent, United States). The analysis of peak-heights in agarose gel electropherogram was shown in Supplementary Figure S2. Finally, the libraries were sequenced on the Illumina NovaSeq platform to obtain 250 bp of paired-end sequences.

#### 2.9.3 Sequencing Data Analysis

The raw sequencing data were spliced using FLASH (Version 1.2.7, http://ccb.jhu.edu/software/FLASH/). After quality control, the effective tags were obtained. The tags were clustered using Uparse software (Version 7.0.1001, http://drive5.com/uparse/) at 97% similarity to obtain operational taxonomic units (OTUs) (Edgar, 2013). The SILVA reference database (Version 138, http://www.arb-silva.de/) based on the Mothur the algorithm, was used to annotate the OTUs with taxonomic information (Quast et al., 2012). Multiple sequence comparisons were conducted using MUSCLE software (Version 3.8.31, http://www.drive5.com/muscle/) (Edgar, 2004). The abundance of OTUs was normalized using the sequence number corresponding to the sample with the smallest sequence. Αlpha-diversity indicator and beta-diversity analysis were subsequently performed. The Wilcoxon Rank-Sum test was used for statistical difference analysis between groups of diversity indicators. The Kruskal–Wallis rank-sum test was used (Games–Howell test was chosen as the post-hoc test) in conjunction with the multiple testing method of false discovery rate to screen for different bacteria. *p* < 0.05 after false discovery rate (FDR) correction was considered to be statistically significant. Finally, data analysis using Phylogenetic Investigation of Communities by Reconstruction of Unobserved States database (PICRUSt) was performed to predict the relevant biological pathways that may be affected by each group exhibiting different microbiota.

### 2.10 Metabolomics Analysis

#### 2.10.1 Serum Sample Processing

A 100 μl of serum sample was added to 400 μl of 80% methanol. The mixture was vortexed, shaken, and centrifuged at 15,000 g for 20 min at 4°C in an ice bath. After centrifugation, the supernatant was diluted with ultrapure water to 53% methanol and centrifuged again at 15,000 g for 20 min at 4°C. The supernatant was collected and used as the test sample. All samples were mixed in equal amounts as a quality control (QC) sample and analyzed periodically throughout the analysis process to ensure the stability and accuracy of the measurement throughout the analysis.

#### 2.10.2 Conditions of Chromatography and Mass Spectrometry

Chromatography was performed on a Hypesil Gold column (C_18_) column (2.1 mm × 100 mm, 1.9 μm) with a mobile phase consisting of (A) 0.1% formic acid and (B) methanol, using a gradient elution of 0 min, 98% A, 2% B; 1.5 min, 98% A, 2% B; 12 min, 0% A, 100% B; 14 min, 0% A, 100% B; 14.1 min, 98% A, 2% B; 17 min, and 98% A, 2% B. The column temperature was set to 40°C with a flow rate of 0.2 ml/min and an injection volume of 2 μl. MS conditions were simultaneous detected in positive- and negative-ion modes using electrospray ionization (ESI). The settings of the ESI were as follows: Spray Voltage: 3.2 kV; Sheath gas flow rate: 40 arb; Aux Gas flow rate: 10 arb; Capillary Temp: 320°C. QC samples were injected every six samples throughout the analytical run, and the data obtained were used to evaluate stability.

#### 2.10.3 Data Processing and Analysis

Molecular signature peaks in the samples were detected based on the results of high-resolution mass spectrometry. The molecular peaks were matched and identified by combining the high-quality mzCloud (https://www.mzcloud.org/), mzVault, and MassList databases constructed from the standards. The raw files (.raw) obtained by MS were imported into Compound Discoverer 3.1 (CD3.1, Thermo Fisher) software for data preprocessing. First, the data were briefly screened by the parameters of retention time and mass-to-charge ratio, and then the peaks from different samples were aligned according to the retention time deviation of 0.2 min and the mass deviation (Part per million, ppm) of 5 ppm to make identification accurate. Subsequently, the data were aligned according to the settings of 5 ppm, signal intensity deviation of 30%, signal-to-noise ratio (S/N) of 3, minimum signal intensity of 1,00,000. Adduct ions for peak extraction and peak area were also quantified. The metabolites were identified by the molecular formula prediction using molecular ion peaks and fragment ions, and were compared with the mzCloud and mzVault and MassList databases. Metabolites with a coefficient of variation of less than 30% in the QC samples were then retained as the final identification results for subsequent analysis. The peaks detected in the samples were integrated using CD3.1 software, where the peak area of each characteristic peak represented the relative quantitative values of a metabolite, and the quantitative results were normalized using the total peak area to obtain the final quantitative results for the metabolites. The data were then subjected to QC to ensure the accuracy and reliability of the results.

Next, the metabolites were subjected to a multivariate statistical analysis, including principal component analysis (PCA) and orthogonal partial least squares discriminant analysis (OPLS-DA), to identify differences in the metabolic patterns among groups. PCA and OPLS-DA were performed using SIMCA software (version 14.1, Umetrics, Sweden). Furthermore, two-tailed Student’s *t*-test was used to analyze the normalized peak areas for each metabolite. Differential metabolites between Control and Model groups and between Model and SHYS high-dose groups were screened based on *p* < 0.05 and variable importance of projection (VIP) > 1. Finally, the biological significance of metabolite correlations was explained by functional analysis including metabolic pathways. Metabolic pathway enrichment analysis was performed for differential metabolites with a fold-change (FC) > 1.20 or FC < 0.80 based on MetaboAnalyst software (https://www.metaboanalyst.ca/) and Kyoto Encyclopedia of Genes and Genomes (KEGG) database (https://www.kegg.jp/).

#### 2.10.4 Statistical Methods

The experimental data were analyzed using SPSS 20.0 statistical software. Data were expressed as the mean ± standard deviation (SD). *t*-test was used for comparison between two groups. One-way analysis of variance and post-hoc analysis was used for comparison among multiple groups. Differences were considered statistically significant at *p* < 0.05.

## 3 Results

### 3.1 Identification of Main Compounds in SHYS by UPLC-MS Analysis

Ferulic acid, atractylodin, tanshinone, astragaloside, hyperoside, loganin, gallic acid, morroniside, allantoin, oleanic acid, ginsenoside Rb1, ginsenoside Re, ginsenoside Rg1, paeoniflorin, vaccarin, pyrrolidinium, leonurine, and catalpol were used as the reference standards to validate the main compounds in SHYS. The detailed information of these compounds were shown in Supplementary Figure S3. The typical based peak intensity (BPI) chromatograms of SHYS and these reference standards were shown in Supplementary Figure S4. The characteristic fragment ions of these compounds were shown in Supplementary Table S1. Astragaloside in *Astragalus mongholicus* Bunge, ginsenoside Rb1, ginsenoside Re, and ginsenoside Rg1 in *Panax quinquefolius* L., allantoin in *Dioscorea oppositifolia* L., loganin and morroniside in *Cornus officinalis* Siebold and Zucc., hyperoside in *Cuscuta chinensis* Lam., Pyrrolidinium in *Polygonatum sibiricum* Redouté, Catalpol in *Rehmannia glutinosa* (Gaertn.) DC., gallic acid in *Euryale ferox* Salisb., oleanic acid in *Rosa laevigata* Michx., leonurine in *Leonurus japonicus* Houtt., tanshinone in *Salvia miltiorrhiza* Bunge, ferulic acid in *Conioselinum anthriscoides* ‘Chuanxiong’, atractylodin in *Atractylodes lancea* (Thunb.) DC., paeoniflorin in *Paeonia lactiflora* Pall., and vaccarin in *Gypsophila vaccaria* (L.) Sm. were identified as the preeminent compounds in SHYS.

### 3.2 Effect of SHYS on Body Weight, FBG, Renal Function, and Pathological Changes in DN Rats

**FIGURE 2:**
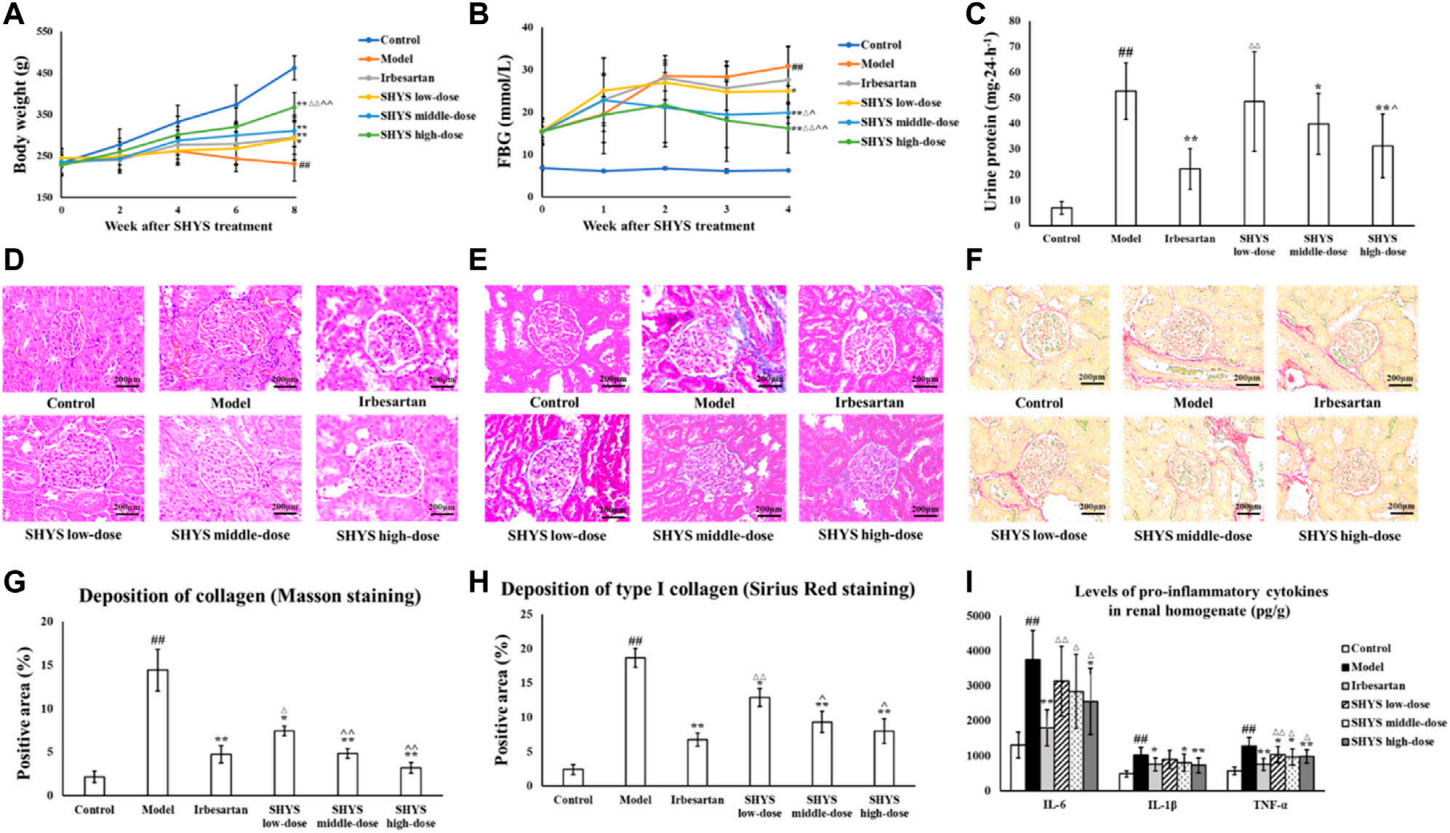
SHYS treatment alleviated the body weight loss, hyperglycemia, proteinuria, pathological changes in kidney and inflammatory response in DN rats. **(A)** SHYS treatment alleviated the body weight loss in DN model rats. **(B)** SHYS treatment decreased the FBG in DN rats. **(C)** SHYS treatment decreased the level of 24-h urine protein. **(D)** H and E staining indicated that SHYS treatment significantly improved the pathological changes of kidney in DN rats (200 ×) **(E–H)** Masson **(E,G)** and Sirius red **(F,H)** staining indicated that SHYS treatment significantly decreased collagen depositions in kidney (200 ×). **(I)** SHYS treatment decreased the levels of pro-inflammatory cytokines in renal tissue homogenates. Control, Model, Irbesartan, SHYS low-dose, SHYS middle-dose and SHYS high-dose (*n* = 10 per group) groups. Data are presented as the mean ± SD. ^##^: *p* < 0.01 as compared to the Control group; ^*^: *p* < 0.05 as compared to the Model group; ^**^: *p* < 0.01 as compared to the Model group; ^△^: *p* < 0.05 as compared to the Irbesartan group; ^△△^: *p* < 0.01 as compared to the Irbesartan group; ^: *p* < 0.05 as compared to the SHYS low-dose group; ^^: *p* < 0.01 as compared to the SHYS low-dose group

At the end of drug administration, the body weight in the Model group decreased significantly compared with that of the Control group (*p* < 0.01). The body weight of the rats in the Irbesartan, SHYS low-, middle-, and high-dose groups increased significantly compared with that of the Model group (*p* < 0.01, *p* < 0.05, *p* < 0.01, *p* < 0.01, respectively, Figure 2A). FBG levels were ≥12 mmol/L in all rats 2 weeks after STZ injection. After 8 weeks of SHYS intervention, FBG levels were significantly higher in Model group compared with the Control group (*p* < 0.01) and were lower in the SHYS low-, middle-, and high-dose groups compared with that of the Model group (*p* < 0.05, *p* < 0.01, *p* < 0.01, Figure 2B). The results of renal function tests showed that the serum levels of Cr and BUN as well as 24-h urine protein levels were significantly higher in the Model group compared with that of the Control group (*p* < 0.01, respectively). The intervention with irbesartan and high-dose SHYS significantly reduced the serum levels of Cr (*p* < 0.05, *p* < 0.01, respectively), BUN (*p* < 0.05, *p* < 0.01, respectively), and 24-h urine protein in the DN rats (*p* < 0.01, respectively). The middle-dose SHYS resulted in significantly reduced 24-h urine protein (*p* < 0.05) and serum BUN levels in DN rats (*p* < 0.01, Table 1 and Figure 2C).

**TABLE 1:** Changes of serum Cr and BUN levels in DN rats after SHYS treatment.

Group	Cr (μmol/L)	BUN (mmol/L)
Control	37.39 ± 17.17	3.49 ± 0.2
Model	80.41 ± 32.13^a^	6.82 ± 0.81^a^
Irbesartan	40.73 ± 24.01^b^	5.31 ± 1.48^b^
SHYS low-dose	65.41 ± 32.01^c^	5.55 ± 1.78
SHYS middle-dose	53.83 ± 15.07	3.76 ± 0.84^d^ ^,^ ^c^
SHYS high-dose	39.04 ± 18.19^d^	3.61 ± 0.40^d^ ^,^ ^e^

Control, Model, Irbesartan, SHYS low-dose, SHYS middle-dose and SHYS high-dose (*n* = 10 per group) groups. Data are presented as the mean ± SD.

a *p* < 0.01 as compared to the Control group.

b *p* < 0.05 as compared to the Model group.

c *p* < 0.05 as compared to the Irbesartan group.

d *p* < 0.01 as compared to the Model group.

e *p* < 0.01 as compared to the Irbesartan group.

H and E staining results of the kidneys showed that the glomeruli and tubules of the animals in the Control group had normal structure. No mesentery or mesangial stromal hyperplasia was observed and no inflammatory cell infiltration was evident. The Model group showed focal tubular degeneration and atrophy, slight thickening of the glomerular basement membrane and mesangial hyperplasia. Compared with the Model group, the lesions were reduced in each drug-treated group and the improvement was more significant in the irbesartan group as well as the SHYS middle- and high-dose groups (Figure 2D). Masson staining revealed that the collagen appeared as a blue color by light microscopy. As shown in Figures 2E,G, the glomeruli and tubules of animals in the Control group had normal structures and collagen expression was unchanged. In the Model group, however, the collagen areas in the glomerular mesangial region and basement membrane were showed significantly increased compared with the Control group (*p* < 0.01). The glomerular and tubulointerstitial collagen deposition was lower in Irbesartan, SHYS low-dose, SHYS middle-dose, and SHYS high-dose groups compared with those in the Model group (*p* < 0.01, *p* < 0.05, *p* < 0.01, *p* < 0.01, respectively). The results of Sirius red staining was shown in Figures 2F,H and indicated that the Model group had significant increase of renal interstitial and vascular wall collagen depositions and thickened glomerular arterioles compared with the Control group, whereas the collagen deposition was lower in Irbesartan, SHYS low-dose, SHYS middle-dose, and SHYS high-dose groups compared with those in the Model group (*p* < 0.01, *p* < 0.05, *p* < 0.01, *p* < 0.01, respectively).

### 3.3 Effect of SHYS on Oxidative Stress and Inflammatory Factor Levels in DN Rats

We further evaluated the effects of SHYS on oxidative stress in DN rats by measuring SOD, MDA, and GSH-Px in renal tissue homogenates from each group. Compared with the Control group, the activities of SOD and GSH-Px (*p* < 0.01, respectively) in the renal tissue homogenates of animals in Model group were significantly decreased, whereas the level of MDA (*p* < 0.01) was significantly increased. Irbesartan, SHYS middle-dose, and SHYS high-dose treated animals exhibited a significant increase in SOD (*p* < 0.01, *p* < 0.05, *p* < 0.01, respectively) and GSH- Px (*p* < 0.01, *p* < 0.05, *p* < 0.01, respectively) activities and decreased MDA (*p* < 0.01, *p* < 0.05, *p* < 0.01, respectively) levels in renal tissue homogenates (Table 2). The effects of SHYS on the inflammatory response of DN rats was also determined by measuring the levels of pro-inflammatory factors, IL-6, IL-1β, and TNF-α, in renal tissue homogenates from each group by ELISA. The levels of proinflammatory factors, IL-6, IL-1β, and TNF-α (*p* < 0.01, respectively) in renal tissue homogenates were significantly increased in rats of the Model group compared with that of the Control group. The levels of IL-6 in the renal tissue homogenates of DN rats were significantly decreased following treatment with irbesartan and high-dose of SHYS (*p* < 0.01 and *p* < 0.05, respectively). The levels of IL-1β were significantly decreased in Irbesartan, SHYS middle-dose and SHYS high-dose groups compared with the Model group (*p* < 0.05, *p* < 0.05 and *p* < 0.01, respectively). The levels of TNF-α were lower in Irbesartan, SHYS low-dose, SHYS middle-dose and SHYS high-dose groups compared with that in the Model group (*p* < 0.01, *p* < 0.05, *p* < 0.05, *p* < 0.01, respectively, Figure 2I). These results indicated that SHYS exhibits therapeutic effects on DN rats and the efficacy is most significant at high doses. Therefore, high-dose SHYS was selected for subsequent gut microbiota and metabolomics studies.

**TABLE 2:** Changes of SOD, GSH-Px activities and MDA level in renal tissue homogenates after SHYS treatment.

Group	SOD (U/mgprot)	MDA (nmol/mgprot)	GSH-px (U/ mgprot)
Control	184.65 ± 32.81	3.56 ± 0.82	82.07 ± 11.3
Model	115.8 ± 37.01^a^	15.29 ± 3.27^a^	36.65 ± 18.52^a^
Irbesartan	163.2 ± 31.64^b^	5.11 ± 1.87^b^	74.85 ± 11.98^b^
SHYS low-dose	117.61 ± 37.42^c^	14.06 ± 5.78^d^	49.76 ± 15.39^d^
SHYS middle-dose	148.55 ± 27.43^e^	11.09 ± 2.81^d^ ^,^ ^e^	56.63 ± 21.64^c^ ^,^ ^e^
SHYS high-dose	167.89 ± 26.99^b^ ^,^ ^f^	8.25 ± 2.05^b^ ^,^ ^d^ ^,^ ^g^	61.62 ± 14.15^b^ ^,^ ^c^

Control, Model, Irbesartan, SHYS low-dose, SHYS middle-dose and SHYS high-dose (*n* = 10 per group) groups. Data are presented as the mean ± SD.

a *p* < 0.01 as compared to the Control group.

b *p* < 0.01 as compared to the Model group.

c *p* < 0.05 as compared to the Irbesartan group.

d *p* < 0.01 as compared to the Irbesartan group.

e *p* < 0.05 as compared to the Model group.

f *p* < 0.01 as compared to the SHYS low-dose group.

g *p* < 0.05 as compared to the SHYS low-dose group.

### 3.4 Effects of SHYS on Gut Microbiota in DN Rats

**FIGURE 3:**
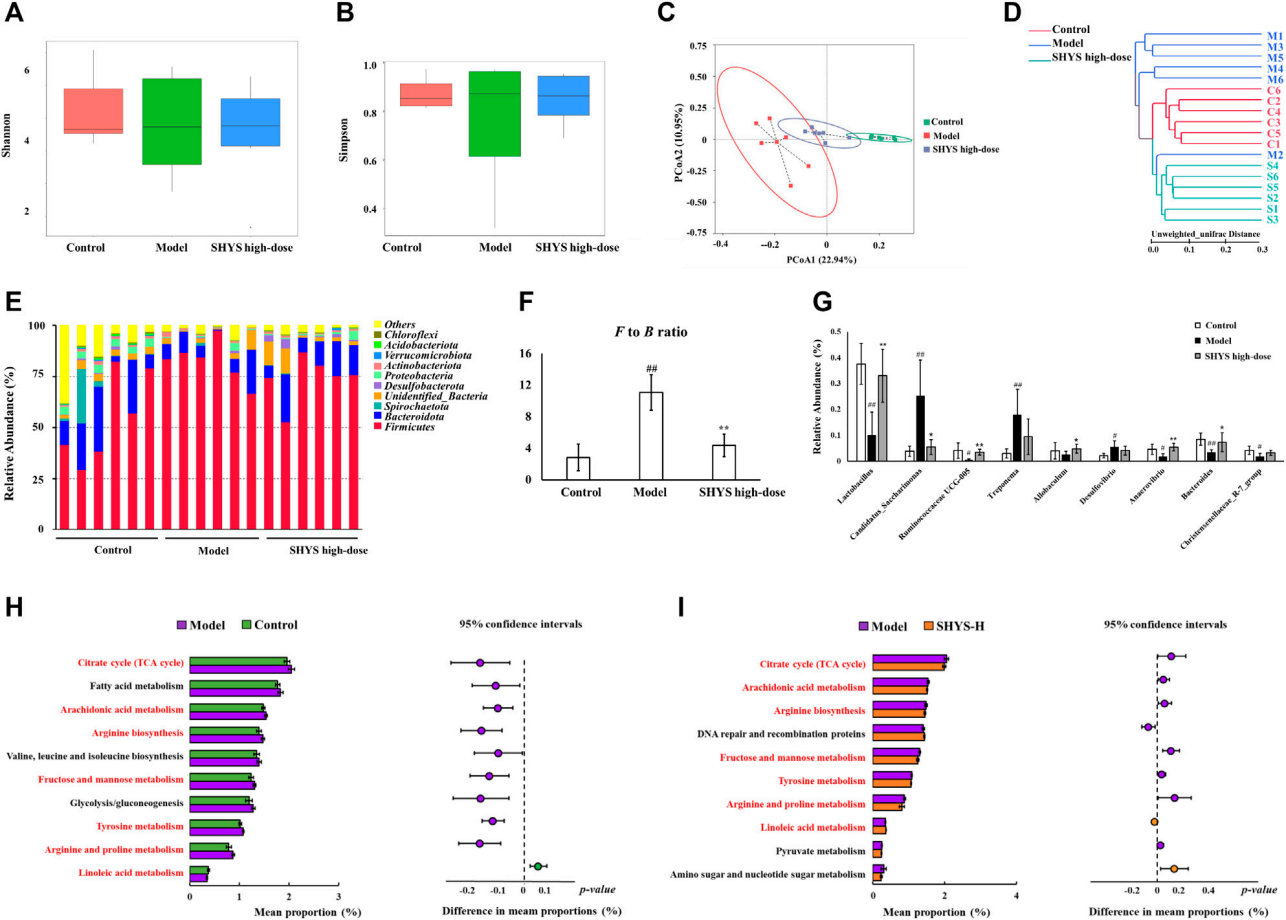
SHYS treatment affected the diversity and abundances of gut microbiota in DN rats **(A,B)** No significant differences were observed among the Control, Model and SHYS high-dose groups. **(C,D)** PCoA and system clustering tree indicated more similar beta diversity between SHYS high-dose and Control groups than that between the Model and Control groups (C: Control group; M: Model group; S: SHYS high-dose group). **(E,F)** At the phylum level, SHYS treatment decreased the *F* to *B* ratio in DN rats. **(G)** At the genus level, SHYS treatment affected the relative abundances of *Lactobacillus*, *Ruminococcaceae UCG-005*, *Allobaculum*, *Anaerovibrio*, *Bacteroides* and *Candidatus_Saccharimonas* in DN rats **(H,I)** The differential metabolic pathways (written in red) of SHYS on DN were predicted using PICRUSt analysis based on the 16S rRNA sequencing data. Control, Model and SHYS high-dose (*n* = 6 per group) groups. Data are presented as the mean ± SD. ^#^: *p* < 0.05 as compared to the Control group; ^##^: *p* < 0.01 as compared to the Control group; ^*^: *p* < 0.05 as compared to the Model group; ^**^: *p* < 0.01 as compared to the Model group

We used 16S rRNA high-throughput sequencing to determine the changes in gut microbiota of DN rats after SHYS treatment. The abundance and diversity of the microbial communities within the samples were analyzed using alpha diversity (i.e., Shannon index and Simpson index). The results indicated that the Shannon and Simpson indexes did not reveal significant differences in the Model group compared with the Control group. Similarly, there was no significant difference in the Shannon and Simpson indexes in the SHYS high-dose group compared with the Model group (Figures 3A,B). Next, we analyzed the composition of microbial communities of different samples using beta diversity and evaluated them by principal coordinate analysis (PCoA) and cluster analysis. The PCoA analysis was performed based on unweighted distance, in which the higher the similarity of species composition structure between samples, the more clustered in the diagram. Conversely, the lower the similarity between samples, the more distant the linear distance. The PCoA results indicated that sample points in the Model group could be completely separated from those in the Control group, and sample points in the SHYS high-dose group were very close to that of the Control group (Figure 3C). The clustering analysis also showed similar results (Figure 3D). The results indicated that the distance from the Control group to the SHYS high-dose group was closer than that between the Control group and the Model group. The above results indicated that the overall structure and composition of the gut microbiota of DN rats changed significantly and high-dose SHYS effectively reversed this change.

The composition of the gut microbiota in each group of samples at the phylum level indicated that *Firmicutes* and *Bacteroidetes* were the dominant species (Figure 3E). The *Firmicutes* to *Bacteroidetes* (*F* to *B*) ratio was significantly higher in the Model group compared with that in the Control group (*p* < 0.01), whereas the *F* to *B* ratio was significantly lower in the SHYS high-dose group compared with that in the Model group (*p* < 0.01, Figure 3F). At the genus level, *Candidatus_Saccharimonas*, *Treponema* and *Desulfovibrio* (*p* < 0.01, *p* < 0.01, *p* < 0.05, respectively) were at relatively higher abundances in Model group compared with the Control group, whereas *Lactobacillus*, *Ruminococcaceae UCG-005*, *Anaerovibrio*, *Bacteroides* and *Christensenellaceae_R-7_group* (*p* < 0.01, *p* < 0.05, *p* < 0.05, *p* < 0.01, *p* < 0.05, respectively) were relatively lower in abundance in Model group compared with the Control group. *Lactobacillus*, *Ruminococcaceae UCG-005*, *Allobaculum*, *Anaerovibrio* and *Bacteroides* (*p* < 0.01, *p* < 0.01, *p* < 0.05, *p* < 0.01, *p* < 0.05) were relatively more abundant and *Candidatus_Saccharimonas* (*p* < 0.05) was relatively less abundant in the SHYS high-dose group compared with the Model group (Figure 3G).

To determine the effect of high-dose SHYS treatment on the function of the gut microbiota in DN rats, bacterial function prediction was performed using PICRUSt analysis. The top 10 metabolic pathways with the highest proportions and with a *p* value less than 0.05 were listed in Figure 3H (Control group vs Model group) and Figure 3I (Model group vs SHYS high-dose group). Proportions of metabolic pathways that were increased in the Model group compared with Control group but decreased in SHYS high-dose group compared with Model group, or vice versa, were considered as differential pathways, including arachidonic acid metabolism, fructose and mannose metabolism, tricarboxylic acid (TCA) cycle, arginine and proline metabolism tyrosine metabolism, linoleic acid metabolism, and arginine biosynthesis pathways.

### 3.5 Effect of SHYS on Serum Metabolite Levels in DN Rats

**FIGURE 4:**
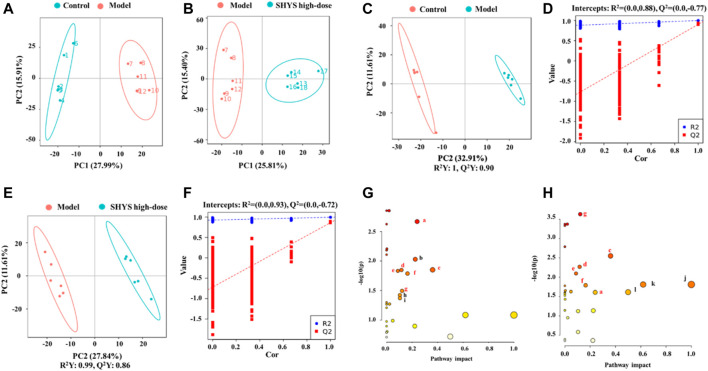
SHYS treatment modulated the serum metabolites in DN rats **(A,B)** Scores plots of PCA between the Control and Model groups **(A)** and between the Model and SHYS high-dose groups **(B–D)** Scores plots of OPLS-DA between the Control and Model groups and the corresponding coefficient of loading plots. **(E,F)** Scores plots of OPLS-DA between the Model and SHYS high-dose groups and the corresponding coefficient of loading plots **(G,H)** Summary of pathway analysis of serum samples between Control and Model groups **(G)** and between Model and SHYS high-dose groups **(H)**, the sames pathways were written in red. a: Glycerophospholipid metabolism; b: Tryptophan metabolism; c: Alanine, aspartate and glutamate metabolism; d: Arginine biosynthesis; e: TCA cycle; f: Tyrosine metabolism; g: Arginine and proline metabolism; h: Cysteine and methionine metabolism; i: Glycerolipid metabolism; j: Phenylalanine, tyrosine and tryptophan biosynthesis; k: Phenylalanine metabolism. Control, Model and SHYS high-dose (*n* = 6 per group) groups.

Liquid chromatography–mass spectrometry (LC-MS) was used to detect and analyze the sera of animals in each group in both positive- and negative-ion conditions to obtain the total ion current of the metabolites. PCA generates new characteristic variables by linearly combining metabolite variables with certain weights and categorizes the data for each group by new principal variables (principal components). The PCA model reflects the original state of the metabolome data, and the degree of aggregation and dispersion of the samples were observed from the PCA model, where the PCA plots showed that the Control group and the Model group were well-differentiated and the Model group and SHYS high-dose group were also well-differentiated (Figures 4A,B). For the identification of differential metabolites, the model of OPLS-DA was employed, and the explanatory rate (*R* ^2^) and predictive power (*Q* ^2^) of the model were evaluated under the established OPLS-DA model. Compared with the Control group, the Model group yielded an *R* ^2^ = 0.88 and a *Q* ^2^ = −0.77 (Figures 4C,D). Compared with the Model group, the SHYS high-dose group had an *R* ^2^ = 0.93 and a *Q* ^2^ = −0.72 (Figures 4E,F). These results indicate that the model is stable and shows good predictive ability.

The *p* < 0.05 and VIP > 1.0 were used on differential metabolites for screening, in which a total of 29 differential metabolites with 13 down-regulated and 16 up-regulated metabolites were screened in the Model group compared with the Control group. A total of 27 differential metabolites including 11 down-regulated and 16 up-regulated metabolites were screened in the SHYS high-dose group compared with the Model group (Table 3). Next, we used MetaboAnalyst software to analyze the metabolic pathways of the identified differential metabolites. Differential pathways were considered with a *p*-value < 0.05 and pathway impact > 0.1. Metabolic pathways that were altered between Control and Model groups included glycerophospholipid metabolism, tryptophan metabolism, alanine, aspartate and glutamate metabolism, arginine biosynthesis, TCA cycle, tyrosine metabolism, arginine and proline metabolism, cysteine and methionine metabolism, and glycerophospholipid metabolism metabolism pathways (Figure 4G). The metabolic pathways altered between SHYS high-dose and Model groups included glycerophospholipid metabolism, tryptophan metabolism, alanine, aspartate and glutamate metabolism, arginine biosynthesis, TCA cycle, tyrosine metabolism, arginine and proline metabolism, arginine and proline metabolism, phenylalanine, tyrosine and tryptophan biosynthesis, phenylalanine metabolism, and D-glutamine and D-glutamate metabolism pathways (Figure 4H). The same metabolic pathway of PICRUSt analysis that had done 16S rRNA sequencing and untargeted metabolomics pathway analysis included arginine biosynthesis, TCA cycle, tyrosine metabolism, and arginine and proline metabolism and were discussed in detail.

**TABLE 3:** The differential metabolites in serum after SHYS treatment.

No	Formula	Rt (min)	m/z	Metabolites	VIP		FC		Trend		Pathway
					M vs C	S vs M	M vs C	S vs M	M vs C	S vs M	
1	C_45_H_82_NO_8_P	15.42	854.59	PC (17:0/20:4)	1.68	1.51	0.48	1.68	↓^##^	↑*	a
2	C_8_H_8_N_2_O_3_	6.91	181.06	Nicotinuric acid	1.39	2.05	1.67	0.57	↑^#^	↓*	—
3	C_3_H_7_N_3_O_2_	1.31	118.06	Guanidineacetic acid	1.35	2.16	1.90	0.56	↑^##^	↓**	g
4	C_10_H_12_N_2_O_3_	8.39	209.09	L-Kynurenine	1.56	2.02	1.80	0.62	↑^##^	↓**	b
5	C_20_H_36_O_5_	11.24	355.25	Prostaglandin F1α	1.06	1.73	0.61	1.70	↓^##^	↑*	—
6	C_4_H_4_O_4_	1.20	115.00	Fumaric acid	1.29	1.45	1.90	0.54	↑^##^	↓**	c,d,e,f
7	C_4_H_9_NO_3_	1.30	118.05	Threonine	1.09	1.22	0.48	1.90	↓^##^	↑**	—
8	C_4_H_9_N_3_O_2_	1.35	132.08	Creatine	1.36	1.82	0.61	1.66	↓^##^	↑*	g
9	C_4_H_6_O_5_	1.20	133.01	D-(+)-Malic acid	1.30	1.50	1.62	0.66	↑^##^	↓**	—
10	C_7_H_15_NO_2_	1.36	146.12	Acetylcholine	1.65	1.94	0.52	1.91	↓^##^	↑**	a
11	C_5_H_11_NO_2_S	1.43	150.06	Methionine	1.57	1.99	1.36	0.65	↑^##^	↓*	h
12	C_5_H_9_NO_2_	1.54	116.07	D-Proline	1.31	1.85	0.55	2.00	↓^##^	↑**	g
13	C_5_H_9_NO_4_	1.21	146.05	L-Glutamic acid	1.18	1.75	1.82	0.56	↑^##^	↓*	c,d,g,i
14	C_5_H_6_O_5_	1.25	145.01	α-Ketoglutaric acid	1.13	2.02	0.45	1.84	↓^##^	↑**	c,d,e,i
15	C_4_H_8_N_2_O_3_	1.24	131.05	Asparagine	1.90	2.41	0.49	2.05	↓^##^	↑**	c
16	C_9_H_17_NO_4_	9.45	204.12	Acetylcarnitine	1.81	2.00	0.66	3.96	↓^#^	↑**	—
17	C_19_H_39_O_7_P	13.04	409.24	Lysopa 16:0	1.38	1.22	1.78	0.67	↑^#^	↓*	a,i
18	C_45_H_80_NO_7_P	15.84	776.56	PE (18:2e/22:4)	1.07	1.35	1.52	0.55	↑^#^	↓**	a
19	C_9_H_11_NO_3_	1.98	180.07	L-Tyrosine	1.51	1.44	1.81	0.47	↑^##^	↓**	f,j,k
20	C_9_H_11_NO_2_	4.54	166.09	L-Phenylalanine	1.82	1.36	1.92	0.43	↑^##^	↓**	j,k
21	C_27_H_44_O3	14.08	415.32	Calcitriol	1.58	1.45	0.44	1.98	↓^#^	↑**	—
22	C_26_H_43_NO_6_	11.15	464.30	Glycocholic acid	1.26	1.49	2.44	0.53	↑^#^	↓*	—
23	C_6_H_9_N_3_O_2_	1.65	154.06	L-Histidine	1.63	1.76	0.47	1.98	↓^##^	↑*	—
24	C_11_H_12_N_2_O_3_	6.55	221.09	5-Hydroxytryptophan	1.17	1.78	1.65	0.61	↑^#^	↓*	b
25	C_26_H_52_NO_7_P	14.77	566.35	LPC 18:1	1.24	1.58	1.73	0.65	↑^##^	↓*	a
26	C_5_H_10_N_2_O_3_	1.32	147.08	L-Glutamine	1.22	1.76	0.76	1.29	↓^#^	↑*	c,d,i
27	C_9_H_8_O_3_	1.98	163.04	Phenylpyruvic acid	1.64	1.29	1.52	0.57	↑^#^	↓**	j,k
28	C_8_H_16_O_2_	10.41	143.11	Caprylic acid	1.37	1.32	0.44	1.71	↓^##^	↑	—
29	C_10_H_19_NO_4_	10.36	218.14	Propionyl-L-carnitine	1.48	2.26	0.59	1.28	↓^##^	↑	—

Control, Model and SHYS high-dose (*n* = 6 per group) groups.

^#^ *p* < 0.05 as compared to the Control group; ^##^ *p* < 0.01 as compared to the Control group; *p < 0.05 as compared to the Model group; **p < 0.01 as compared to the Model group; ↑: content increased; ↓: content decreased; vs: versus; C: control group; M: Model group; S: SHYS high-dose group; Rt: retention time; VIP: variable importance of projection; FC: fold change. a: Glycerophospholipid metabolism; b: Tryptophan metabolism; c: Alanine, aspartate and glutamate metabolism; d: Arginine biosynthesis; e: TCA cycle; f: Tyrosine metabolism; g: Arginine and proline metabolism; h: Cysteine and methionine metabolism; i: Glycerolipid metabolism; j: Phenylalanine, tyrosine and tryptophan biosynthesis; k: Phenylalanine metabolism.

### 3.6 Correlation Analysis of Physiological Data, Oxidative Stress and Inflammatory

#### 3.6.1 Factors, Untargeted Metabolomics and Gut Microbiota

**FIGURE 5:**
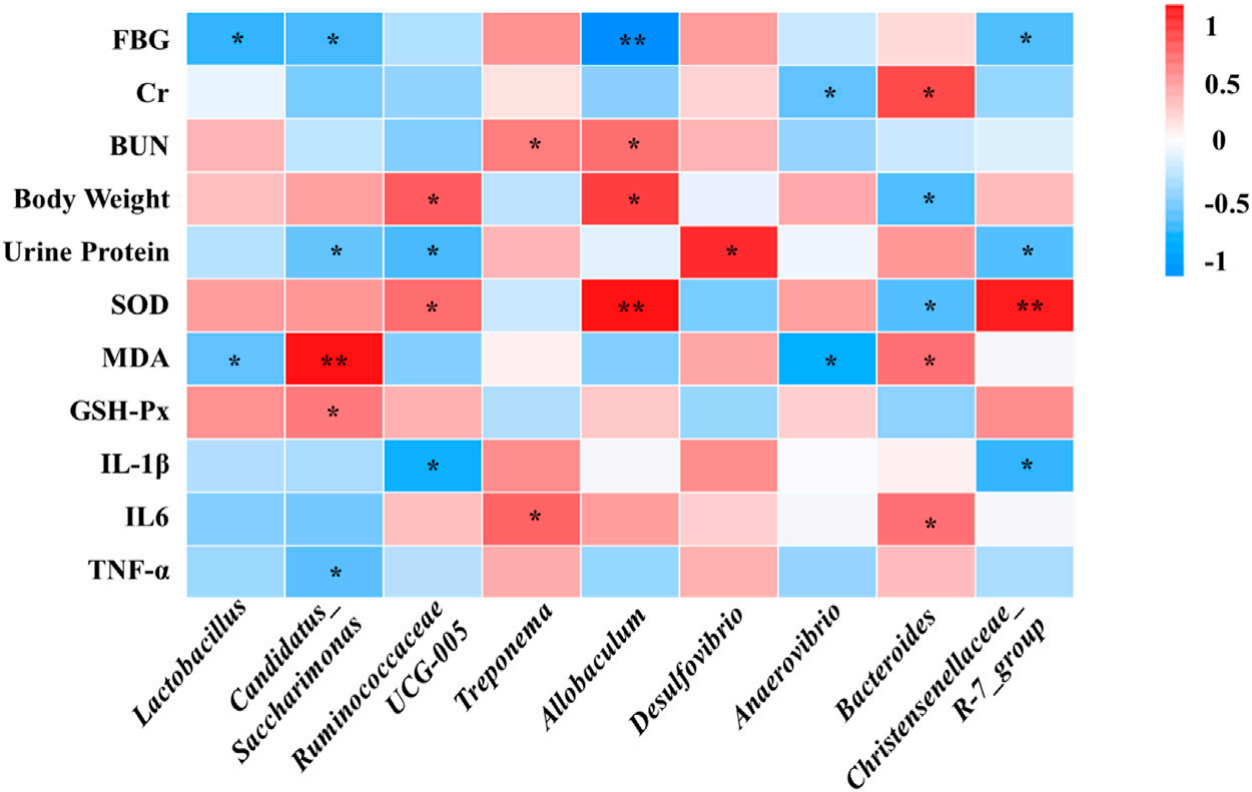
Correlations between between physiological data, oxidative stress and inflammatory factors and gut microbiota were analyzed using spearman’s analysis (heatmap). *X*-axis represents the gut microbiota with differential abundance. *Y*-axis represents the physiological data, oxidative stress and inflammatory factors. The colors of grids represent the correlation analysis value of spearman’s correlation analysis. Grids in red indicate positive correlations (correlation analysis value more than 0.1), while grids in blue indicate negative correlations (correlation analysis value less than −0.1). Color coding scale indicates the correlation analysis value from heatmap, the deeper red or blue indicates higher correlation values. *: *p* < 0.05 between physiological data, oxidative stress and inflammatory factors and gut microbiota. **: *p* < 0.01 between physiological data, oxidative stress and inflammatory factors and gut microbiota.

**FIGURE 6:**
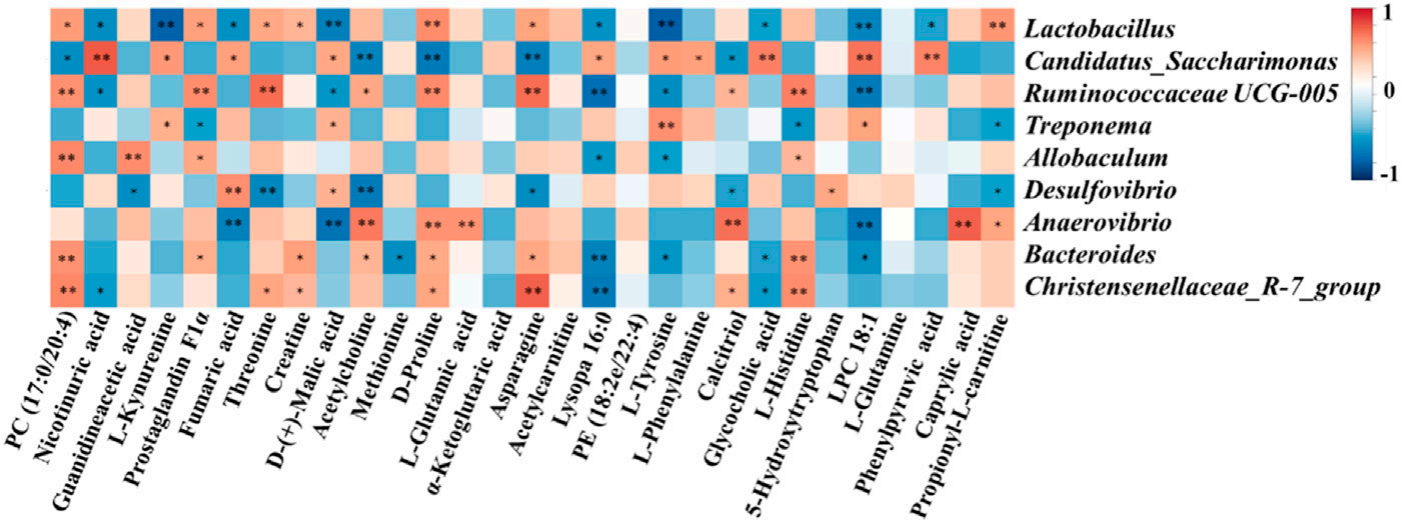
Correlation analysis of untargeted metabolomics and 16S rRNA sequencing. Correlations between between untargeted metabolomics and gut microbiota were analyzed using spearman’s analysis (heatmap). *X*-axis represents the differential metabolites in the serum. *Y*-axis represents the gut microbiota with differential abundance. The colors of grids represent the correlation analysis value of spearman’s correlation analysis. Grids in red indicate positive correlations (correlation analysis value more than 0.1), while grids in blue indicate negative correlations (correlation analysis value less than −0.1). Color coding scale indicates the correlation analysis value from heatmap, the deeper red or blue indicates higher correlation values. *: *p* < 0.05 between differential serum metabolites and gut microbiota. **: *p* < 0.01 between differential serum metabolites and gut microbiota.

Spearman’s correlation analysis was conducted to analyze the relationship between physiological data, oxidative stress and inflammatory factors, differential serum metabolites and gut microbiota at genus level in the Control, Model and SHYS high-dose groups. As shown in Figure 5, *Candidatus_Saccharimona, Ruminococcaceae UCG-005,* and *Christensenellaceae_R-7_group* showed negative correlations and *Treponema and Bacteroides* showed positive correlations with some of the pro-inflamatory cytokines. *Lactobacillus, Candidatus_Saccharimonas, Ruminococcaceae UCG-005, Allobaculum, Anaerovibrio, Bacteroides,* and *Christensenellaceae_R-7_group* showed correlations with some of the oxidative stress factors. In addition, *Lactobacillus, Candidatus_Saccharimonas, Ruminococcaceae UCG-005, Anaerovibrio, Bacteroides,* and *Christensenellaceae_R-7_group* were correlated with most of the metabolites (Figure 6).

## 4 Discussion

In the present study, we used a HFD combined with STZ injection to establish a rat model of DN. Compared with the Control group, the rats in the Model group exhibited a significant increase in FBG levels and there were abnormal biochemical indicators related to renal function, as manifested in increased serum Cr and BUN as well as a significant increase in 24-h urine protein levels. In addition, the pathology results revealed that the renal tissues of the DN model rats showed significant tubular atrophy and glomerular hyperplasia along with a certain degree of inflammatory cell infiltration. This is consistent with the pathological manifestations of DN. The SHYS-treated groups exhibited reduced FBG, improved biochemical indicators of renal function, and alleviated renal histopathological changes to varying degrees in rats with DN. This suggests that SHYS exhibits a therapeutic effect on DN, particularly in the high-dose group. In addition, we selected irbesartan as a positive control drug. The results showed that irbesartan had no significant improvement on blood glucose in rats with DN and there was no significant difference in the improvement of renal function and pathological changes between animals in the positive control group and the high-dose SHYS-treated group. These results suggest that SHYS can be used as an alternative therapy to irbesartan for the treatment of DN.

We further examined the effect of SHYS on inflammation in rats with DN. The hyperglycemic state induces chronic inflammation in the body, which contributes to the infiltration of inflammatory cells in renal tissue and the production of large amounts of pro-inflammatory factors including IL-1β, IL6, and TNF-α (Casqueiro et al., 2012). Our results indicated that the level of pro-inflammatory factors in the renal tissues of rats in the Model group were decreased after SHYS treatment suggesting that SHYS exhibits some anti-inflammatory effects.

We investigated the effect of high-dose SHYS on the gut microbiota of rats in DN model using 16S RNA sequencing technology. There were no significant differences in the Shannon and Simpson indexes between the groups, suggesting that the alpha diversity of gut microbiota in the rat model of DN was unchanged. SHYS did not affect the alpha diversity of gut microbiota in rats with DN. However, in the beta diversity analysis, we found significant differences between animals in the Model group and the Control group in the PCoA plot. The beta diversity of gut microbiota in rats from the high-dose SHYS-treated group was significantly different from that of the Model group. Interestingly, the PCoA plot showed that the beta diversity of gut microbiota in rats from the SHYS-treated groups was more similar to that of the Control group compared with the Model group. These results suggest that SHYS restores the beta diversity of gut microbiota in this DN rat model to a level similar to the Control group. Further analysis of the abundance of microbiota revealed that at the phylum level, the primary species of gut microbiota in the rats from each group were *Firmicutes* and *Bacteroides*. The rats in the DN model showed an increase in the ratio of *F* to *B*, whereas SHYS significantly reduced the *F* to *B* ratio. The elevated *F* to *B* ratio is closely associated with the inflammatory response, metabolic disorders, and other pathological states in DN (Lee et al., 2019). Further analysis at the genus level revealed that SHYS could increase the abundance of *Lactobacillus*, *Ruminococcaceae UCG-005*, *Allobaculum*, *Anaerovibrio* and *Bacteroides* in DN model rats. *Lactobacillus* is a major probiotic with an important role in the regulation of metabolism and immunity (Zhang et al., 2020). A reduced abundance of *Lactobacillus* was found in models of diabetes, fatty liver, and obesity (Mishra et al., 2016; Nova et al., 2016). The metabolic and inflammatory responses of the organism can be improved by the transplantation of *Lactobacillus*. *Ruminococcaceae UCG-005*, *Allobaculum*, and *Bacteroides* produce short-chain fatty acids (SCFAs) by degrading cellulose in food (Zhang et al., 2015; Wu et al., 2020; Xue et al., 2020). SCFAs are important metabolites of the gut microbiota and studies have shown that increasing the SCFA-producing microbiota can significantly improve a number of metabolic diseases, including diabetes and obesity (Yang et al., 2019; Zhang et al., 2019; Birkeland et al., 2020; Tian et al., 2021). In addition, recent studies have shown that SCFAs inhibit the inflammatory responses of renal tissue in DN through G protein-coupled receptors (GPR43 and GPR109A) (Li et al., 2020a). *Anaerovibrio* is capable of catabolizing lipids. Studies have shown that polyphenols in green tea can alleviate chronic inflammation caused by translocation of gut microbiota in an obesity model, while increasing the amount of *Anaerovibrio* in the gut. However, the specific relationship of *Anaerovibrio* to DN remains unknown (Li et al., 2020b). SHYS treatment also caused a decrease of *Candidatus_Saccharimonas* in gut. *Candidatus_Saccharimonas* is a conditional pathogenic bacterium that is significantly elevated in the gut of gout patients (Shao et al., 2017). In addition, green tea leaf powder ameliorates the abnormal lipid metabolism induced by a HFD, while reducing the abundance of *Candidatus_Saccharimonas* in the gut (Li et al., 2020b). Likewise, our correlation analysis also showed negative correlations of *Lactobacillus*, *Candidatus_Saccharimonas*, *Ruminococcaceae UCG-005*, *Allobaculum*, *Anaerovibrio*, *Bacteroides,* and *Christensenellaceae_R-7_grou* with some of the physiological indicies (FBG, Cr, BUN, body weight and Urine Protein), oxidative stress factor (MDA) and pro-inflammatory cytokines (IL-1β, IL-6, TNF-α). Correlation analysis also showed positive correlations of *Treponema* and *Desulfovibrio* with physiological indicies (BUN, Urine Protein) and pro-inflammatory cytokine (IL-6). Further studies should also be carried out using fecal transplantation or gut microbiota depletion models to verify whether SHYS can ameliorate hyperglycemia, hyperlipidemia, IR, oxidative stress and inflammatory responses to treat DN through regulating these gut microbiota.

PCA and OPLS-DA of serum untargeted metabolomics revealed that the metabolites of DN in the rat model were significantly different from those of normal rats. In addition, the serum metabolite levels of rats after SHYS treatment were also significantly different from those of DN rats. Metabolic pathway analysis for differential metabolites using MetaboAnalyst showed that SHYS had an impact on several metabolic pathways including arginine and proline metabolism, alanine, aspartate and glutamate metabolism, arginine biosynthesis, TCA cycle, tyrosine metabolism, and glycerophospholipid metabolism. After correlating the differential metabolic pathways obtained from metabolomics with those deduced from 16s rRNA sequencing, the pathways of arginine biosynthesis, TCA cycle, tyrosine metabolism, and arginine and proline metabolism were the most common, suggesting that SHYS may play a role in the treatment of DN by regulating gut microbiota, which in turn affects arginine biosynthesis, the TCA cycle, tyrosine metabolism, and arginine and proline metabolism.

### 4.1 Arginine Biosynthesis and Arginine and Proline Metabolism

Amino acid metabolism is closely related to metabolic disorders during DN. In the present study, we found that the level of guanidineacetic acid and L-glutamic acid were elevated in DN model rats and significantly decreased after treatment with SHYS. The level of creatine, D-proline, and L-glutamine were decreased and significantly increased after treated with SHYS. Arginine is the largest nitrogen-donating amino acid and is a precursor to proline and creatine (Ge et al., 2020). Proline metabolizes and generates electrons as well as reactive oxygen species, leading to a variety of downstream effects including blocking the cell cycle, autophagy, and apoptosis (Selen et al., 2015). Creatine is a naturally produced nitrogen-containing molecule that promotes the ATP cycle and provides energy to muscles and cells (Arlin et al., 2014). Creatine may reduce damage by inhibiting inflammation, oxidative stress, and aging (Deminice et al., 2016; Aljobaily et al., 2020). Studies have found that supplementation of creatine to diabetic patients results in hypoglycemic effects (Gualano et al., 2011). Guanidineacetic acid is an immediate precursor of creatine biosynthesis, however, this reaction causes elevated levels of homocysteine in the blood. This can result in vasculopathy, which is one of the causes of diabetic macrovascular and microvascular complications in diabetic patients (De Luis et al., 2005; Zhao et al., 2018; Ma et al., 2020). L-glutamic acid and L-glutamine are interconvertible. L-glutamine is an abundant free amino acid inside and outside of human cells that plays an essential role in protein and energy metabolism. It is a precursor substance for the synthesis of amino acids, proteins, and nucleic acids (Tanaka et al., 2015; Kou et al., 2016). Paul et al. found that L-glutamine is effective at resisting obesity and insulin tolerance (Petrus et al., 2020). L-glutamine provides energy to immune cells, such as lymphocytes and macrophages, ensuring immune cell proliferation, and enhancing immunity (Csibi et al., 2014). Also, L-glutamine has anti-inflammatory and antioxidant effects. L-glutamine is a precursor substance for glutathione synthesis and reduces the inflammatory response by increasing glutathione synthesis, inhibiting the NF-κB pathway, and decreasing the levels of IL-8 and TNF-α (Pusapati et al., 2016). Interestingly, the ratio of glutamine to glutamic acid is strongly associated with diabetes and insulin resistance. An elevated ratio is significantly associated with a low risk of developing diabetes (Cheng et al., 2012; Rhee et al., 2018), which primarily occurs through various cell related metabolic events, such as protein synthesis, muscle growth, uropoiesis in the liver, insulin secretion, hepatic and renal gluconeogenesis, neurotransmitter synthesis and glutathione production (Newsholme et al., 2003). A Spearman analysis revealed that guanidineacetic acid was positively correlated with *Allobaculum* and negatively correlated with *Desulfovibrio*; D-Proline was positively correlated with *Lactobacillus, Ruminococcaceae UCG-005, Anaerovibrio, Bacteroides* and *Christensenellaceae*_R-7_group and negatively correlated with *Candidatus_Saccharimona*; creatine was positively correlated with *Lactobacillus, Bacteroides* and *Christensenellaceae_R-7_group*; and L-glutamic acid was positively correlated with *Anaerovibrio.* Therefore, we hypothesize that the effect of SHYS on arginine biosynthesis and arginine and proline metabolism may be related to the regulation of the abundance of *Allobaculum, Desulfovibrio*, *Lactobacillus*, *Ruminococcaceae UCG- 005*, *Anaerovibrio*, *Bacteroides*, *Candidatus_Saccharimona*, and *Christensenellaceae*_R-7_group abundance.

### 4.2 TCA Cycle

The TCA cycle is the final metabolic pathway for carbohydrates, lipids, and amino acids (Chen et al., 2014). It also affects the immune system and is closely associated with various metabolic diseases, including diabetes (Choi et al., 2021). In the present study, we found that fumaric acid was significantly elevated in the DN rat model and decreased after SHYS administration, whereas alpha-ketoglutaric acid was increased after SHYS administration. Fumaric acid is a dicarboxylic acid that is produced by succinic dehydrogenase from precursor adenosine in the TCA cycle and is converted to malic acid by fumarate hydratase (You et al., 2015). The metabolic disorder of fumaric acid may be associated with renal impairment in diabetes, in which its accumulation leads to oxidative stress and prolonged oxidative stress leads to kidney injury in DN (Zheng et al., 2015). Studies have found that the accumulation of fumaric acid is positively associated with progression toward DN in patients (Liu, et al., 2018). Elevated levels of fumaric acid in DN causes endoplasmic reticulum stress and HIF-1α expression, steering metabolism to the glycolytic pathways and leading to pathological renal injury (Isaacs et al., 2005; Tong et al., 2011; Linehan and Rouault, 2013). Alpha-ketoglutaric acid is a key intermediate in the TCA cycle located between succinyl-coenzyme A and isocitric acid. As a key member of the anaplerotic reactions, alpha-ketoglutaric acid regulates ATP production and reduces NAD+/NADH production in the TCA cycle, thereby affecting ROS levels and immune system homeostasis (B Zdzisińska et al., 2017). In addition, alpha-ketoglutaric acid is an important source of glutamine and glutamate, which are required for the synthesis of amino acids and collagen (Xiao et al., 2016). It was found that alpha-ketoglutaric acid supplementation protects mice from myocardial ischemia reperfusion injury (Olenchock et al., 2016). A Spearman analysis showed that fumaric acid was positively correlated with *Candidatus_ Saccharimonas* and *Desulfovibrio*, and negatively correlated with *Lactobacillus and Anaerovibrio.* No bacterial was found significantly corrected with alpha-ketoglutaric acid. Therefore, we hypothesized that the effect of SHYS on the TCA cycle may be associated with the regulating the abundance of *Candidatus_Saccharimonas*, *Desulfovibri, Lactobacillus,* and *Anaerovibrio.*

### 4.3 Tyrosine Metabolism

Tyrosine metabolism is associated with a variety of diseases including fatty liver, insulin resistance, and obesity (Gaggini et al., 2018; Gitto et al., 2018). In the present study, we found that L-tyrosine was significantly elevated in rats of the model group and decreased after the administration of SHYS. Therefore, high levels of tyrosine may promote fatty acid synthesis, which further promotes fat deposition in the liver (Jin et al., 2016). Meanwhile, the characteristic nitrification of tyrosine residues in proteins using peroxynitrite (ONOO-) produces nitrotyrosine (NT). NT is involved in the development of diabetes and its complications and elevated levels of NT can disrupt renal pathology and cause renal dysfunction in diabetic rats (Chew et al., 2010). Increased levels of NT have also been observed in patients with DN (Julius et al., 2008; Castro et al., 2011). NT induces glomerular mesangial cells to express NF-κB, MCP-1, and TGF-β1, causing inflammatory injury and further aggravating nephropathy (Jing et al., 2018). In addition, NT is also a marker of oxidative stress in diabetic patients and NT activates the ERK pathway to increase iNOS and produce excess ROS to mediate oxidative stress damage in the body (Inoguchi and Takayanagi, 2008). A Spearman analysis showed that L-tyrosine was positively correlated with *Candidatus_Saccharimonas* and *Treponema*, and negatively correlated with *Lactobacillus, Ruminococcaceae UCG-005, Allobaculum,* and *Bacteroides*. Therefore, we speculated that the effect of SHYS on the tyrosine metabolism may be with the regulation of the abundance of *Candidatus_Saccharimonas*, *Treponema*, *Lactobacillus, Ruminococcaceae UCG-005, Allobaculum,* and *Bacteroides*.

In conclusion, our study revealed multiple ameliorative effects of SHYS on DN including the alleviation of hyperglycemia and the improvement of renal function, pathological changes in kidney, oxidative stress, and the inflammatory response. The mechanism of SHYS on DN may be related to the improvement of gut microbiota which regulates arginine biosynthesis, TCA cycle, tyrosine metabolism, and arginine and proline metabolism.

## Data Availability

The datasets presented in this study can be found in online repositories. The names of the repository/repositories and accession number(s) can be found below: NCBI (accession: PRJNA778921).
