# Supplementary material for: San-Huang-Yi-Shen Capsule Ameliorates Diabetic Nephropathy in Rats Through Modulating the Gut Microbiota and Overall Metabolism
Source: Front Pharmacol. 2022 Jan 4;12:808867. doi: 10.3389/fphar.2021.808867 (PMC8764181; doi:10.3389/fphar.2021.808867)
Supplement: Supplementary file 1 [file DataSheet1.docx]

**
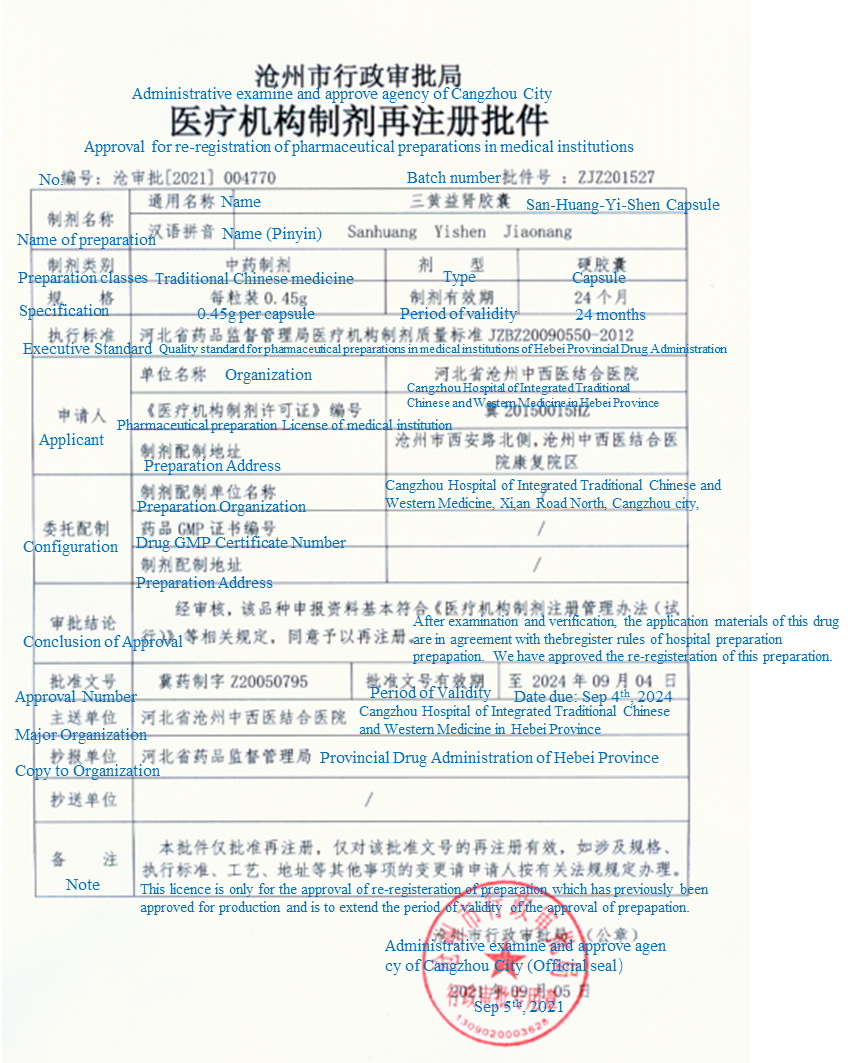
**

**Figure S1:** The production licence of SHYS.

**a**

**
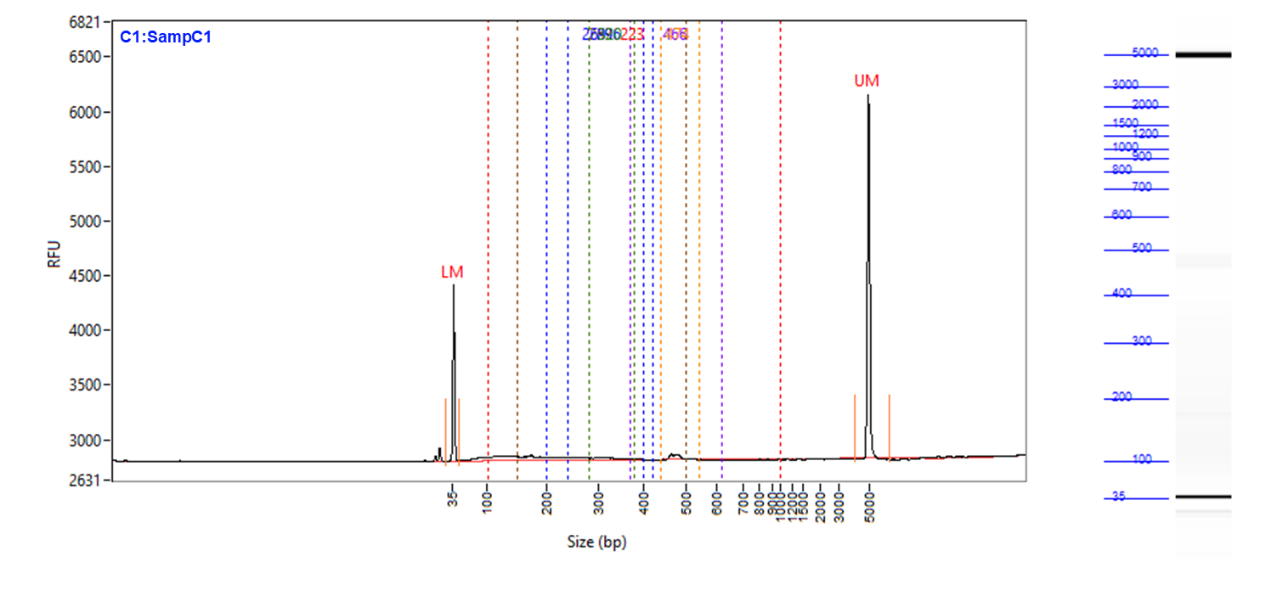
b**

**
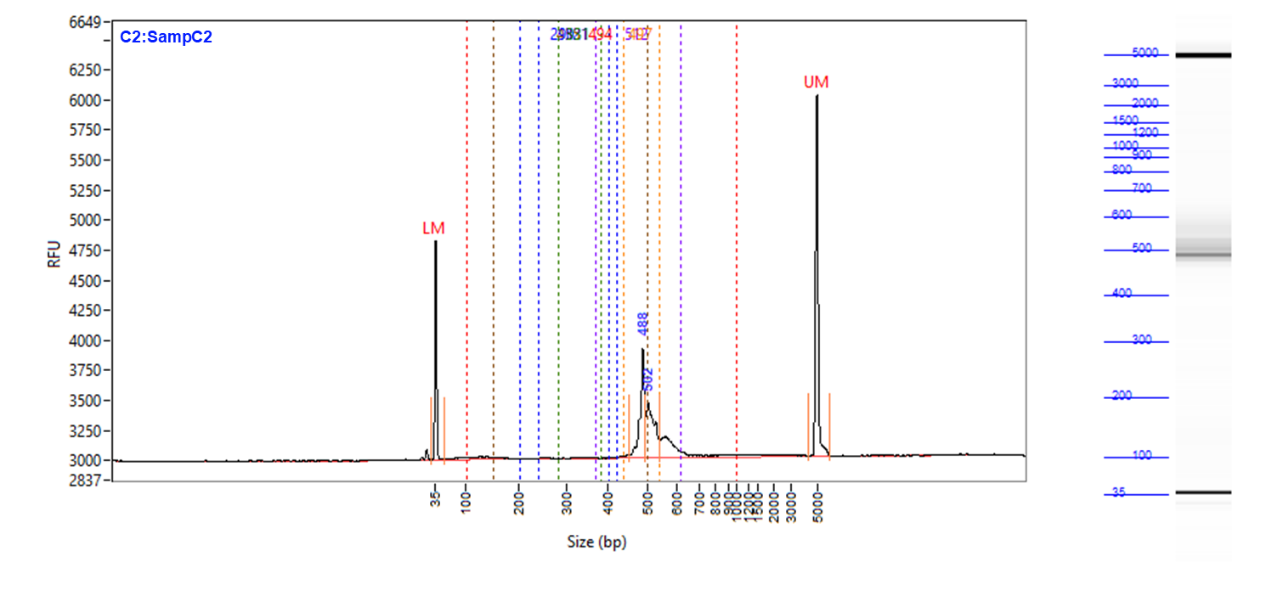
c**

**
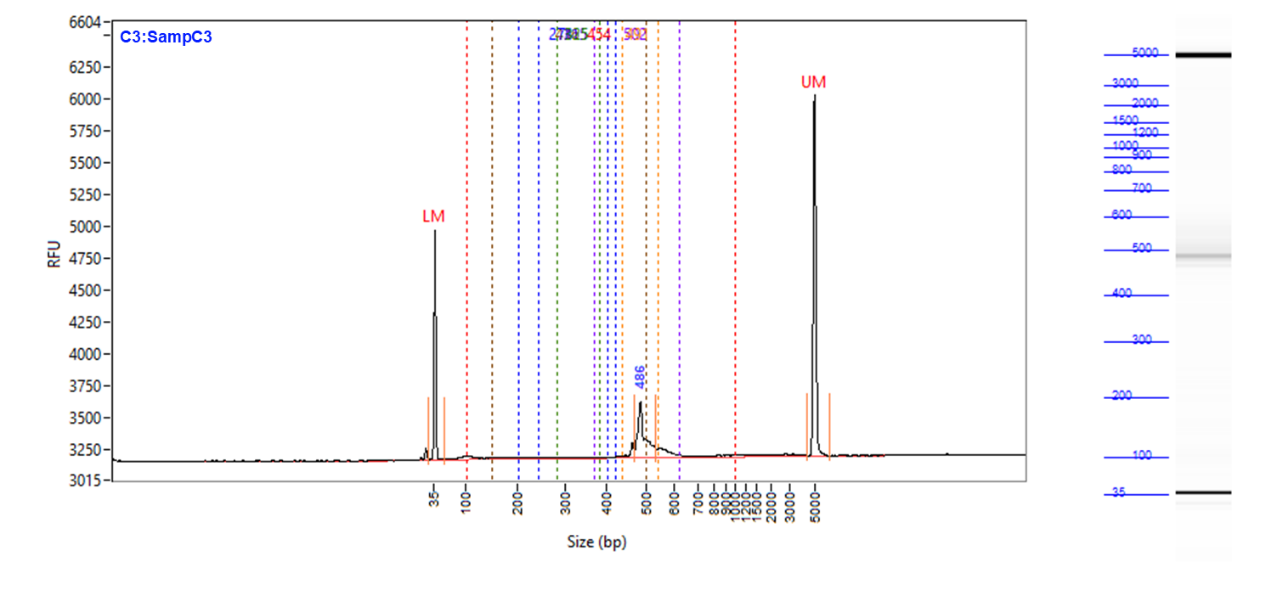
**

**d**

**
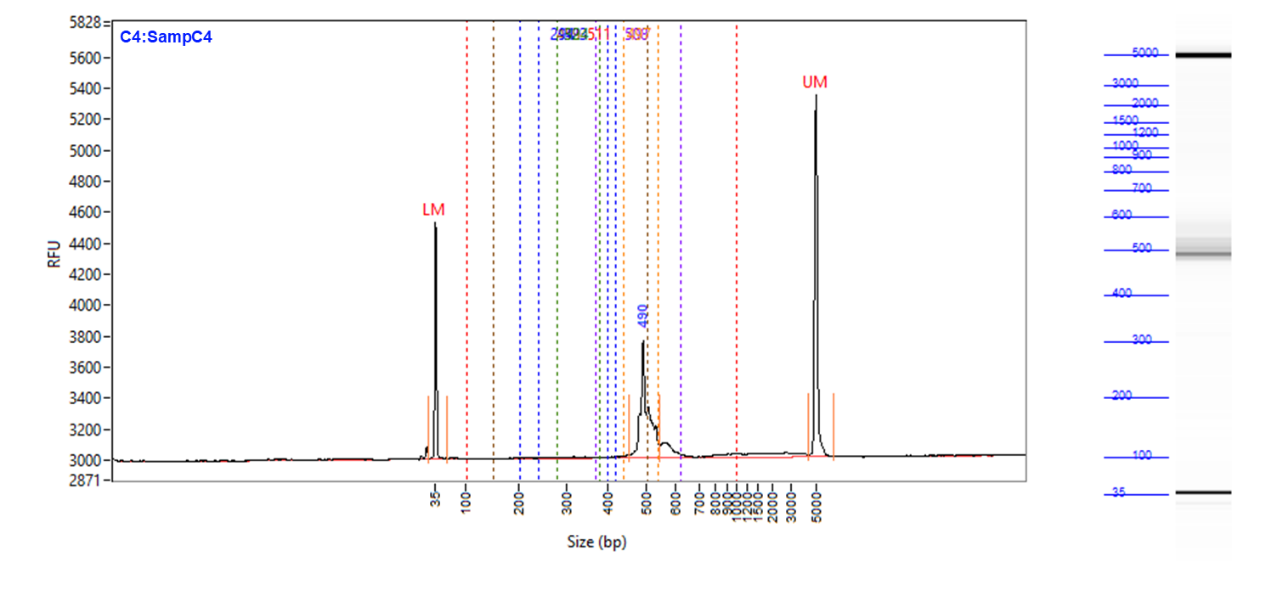
**

**e
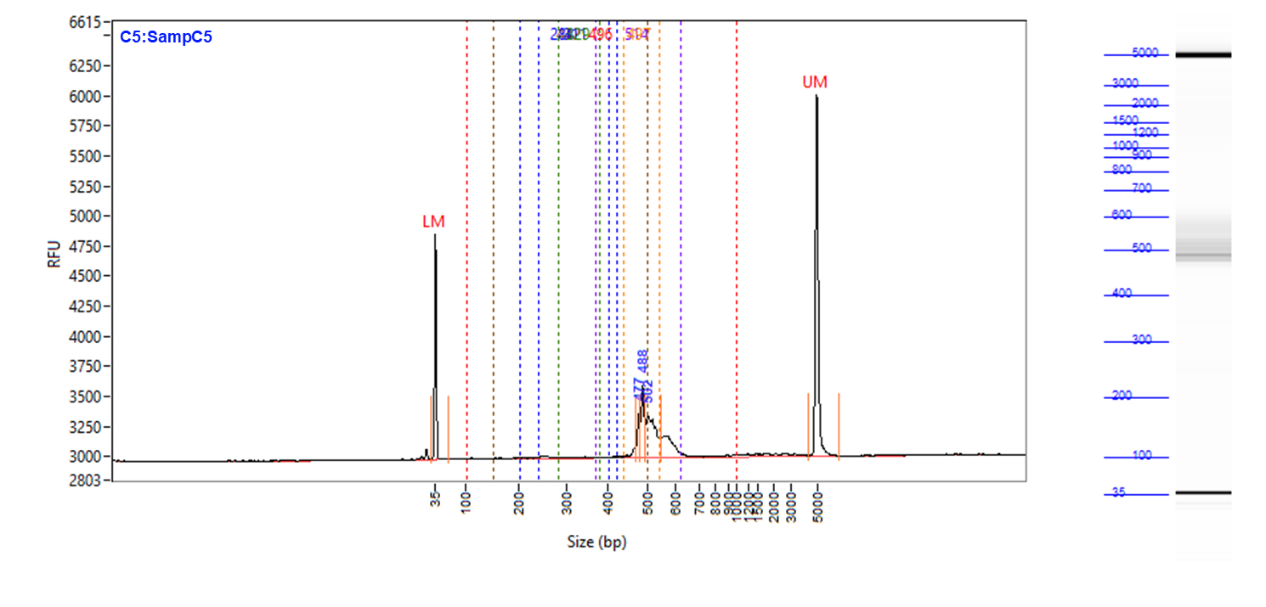
**

**f**

**
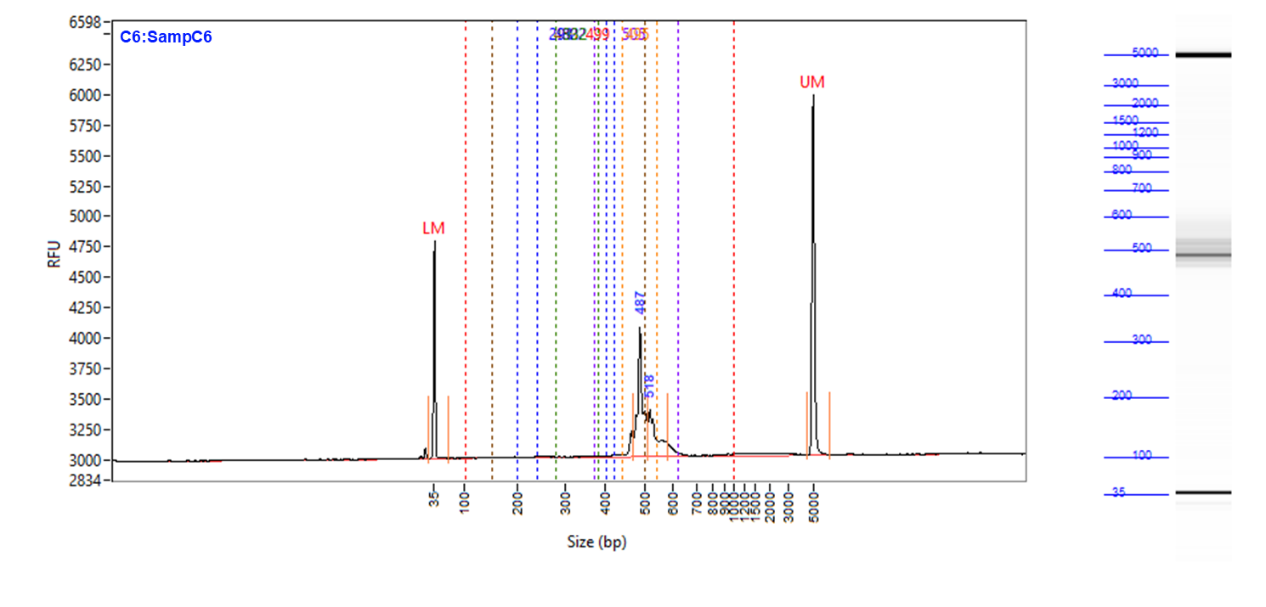
**

**g**

**
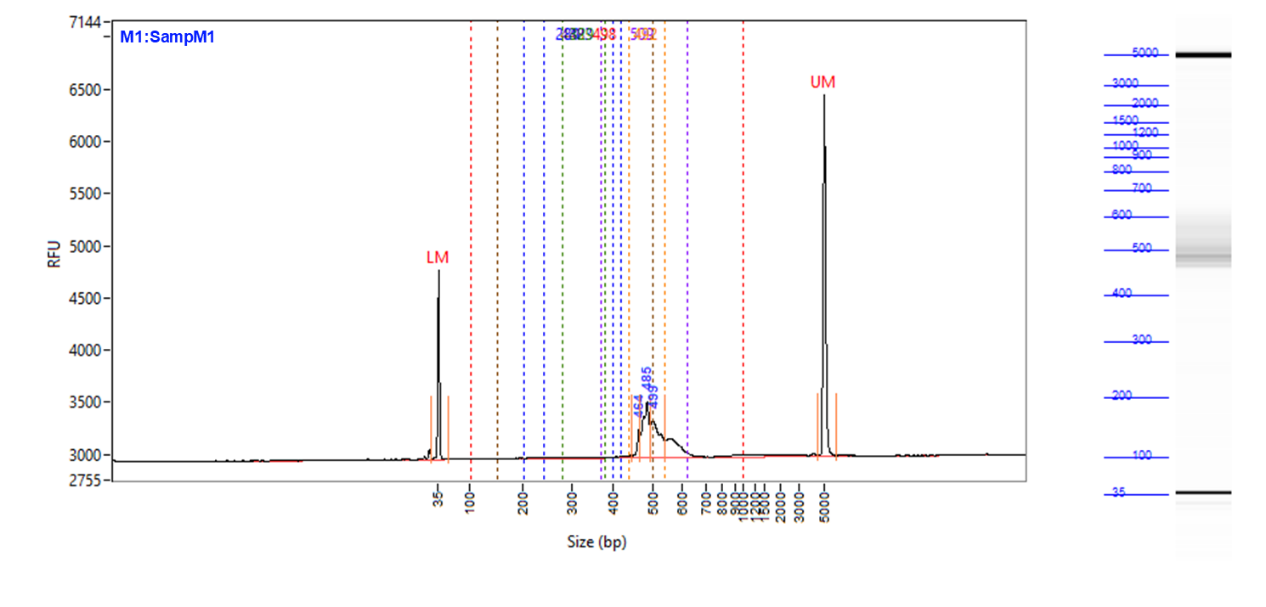
**

**h**

**
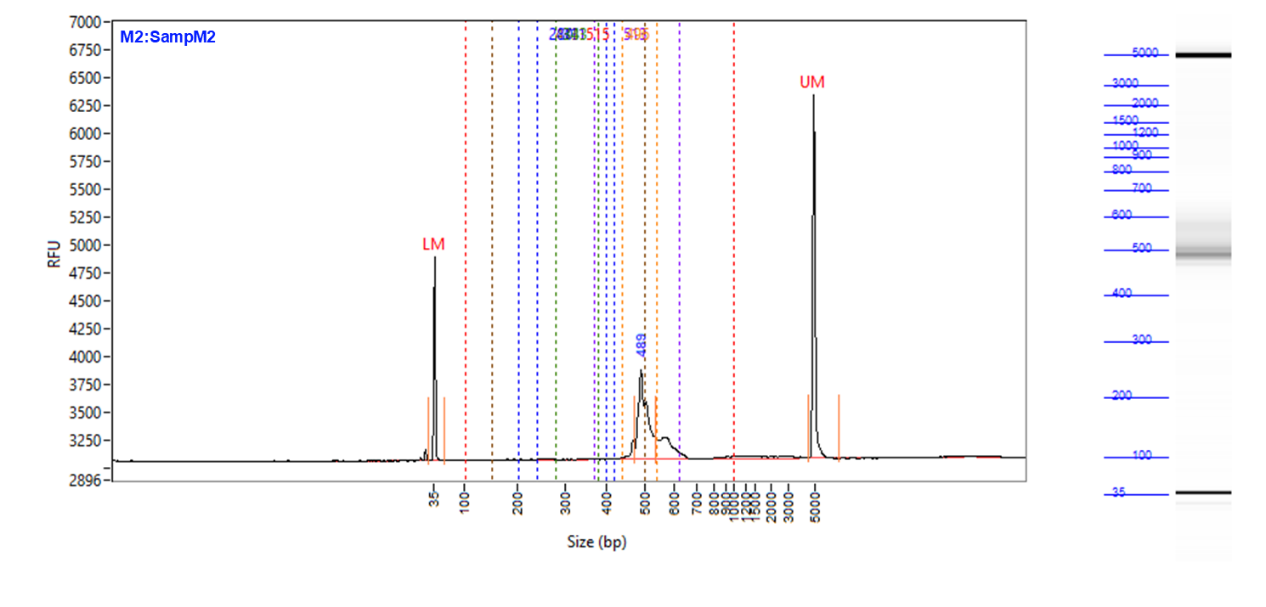
**

**i**

**
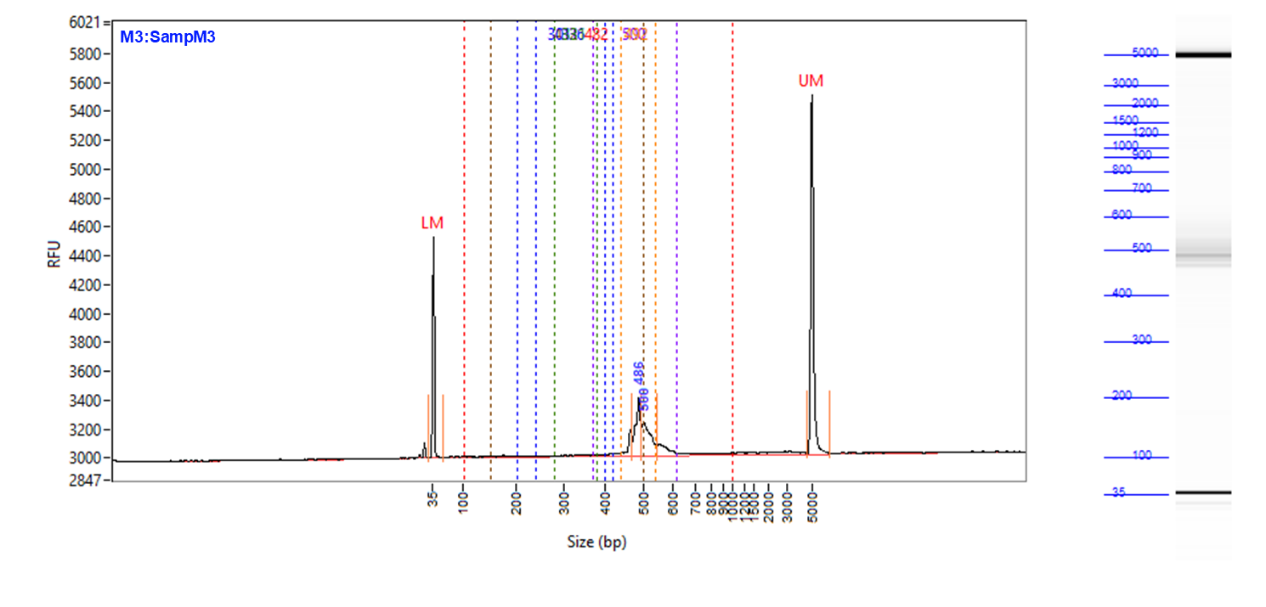
**

**j**

**
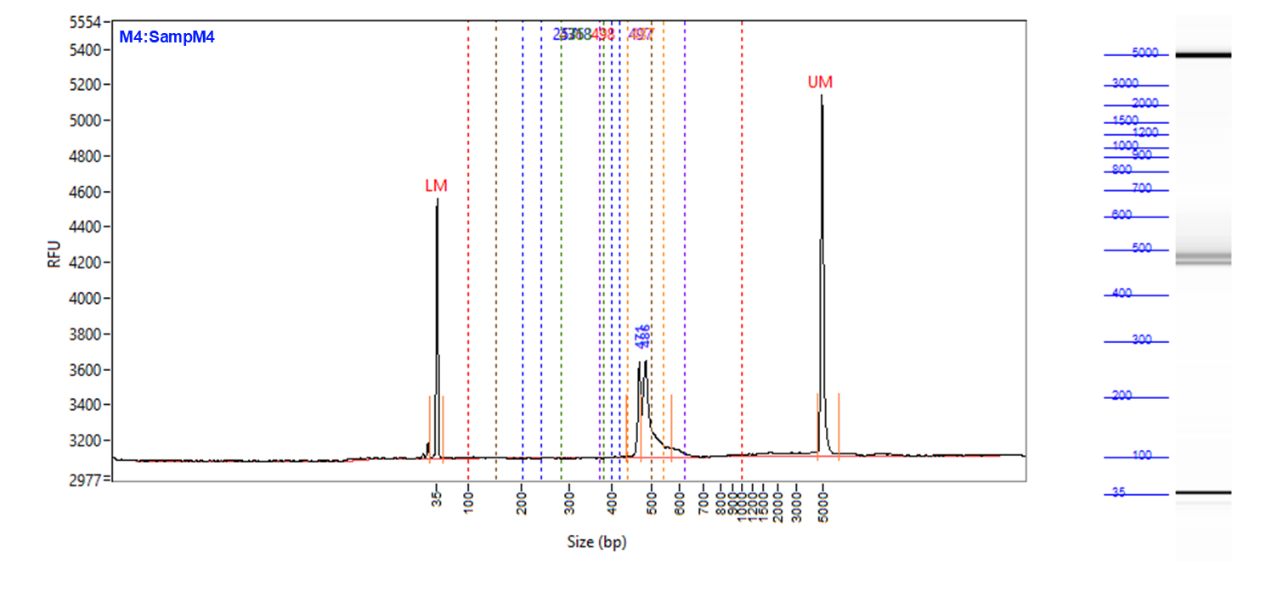
**

**k**

**
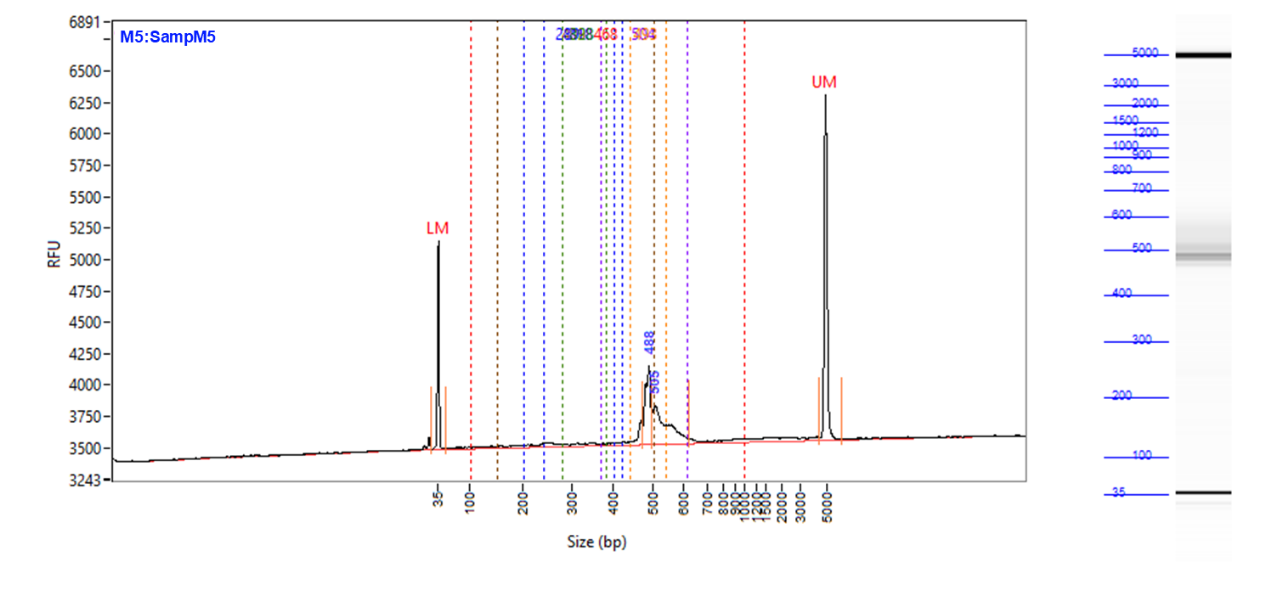
**

**l**

**
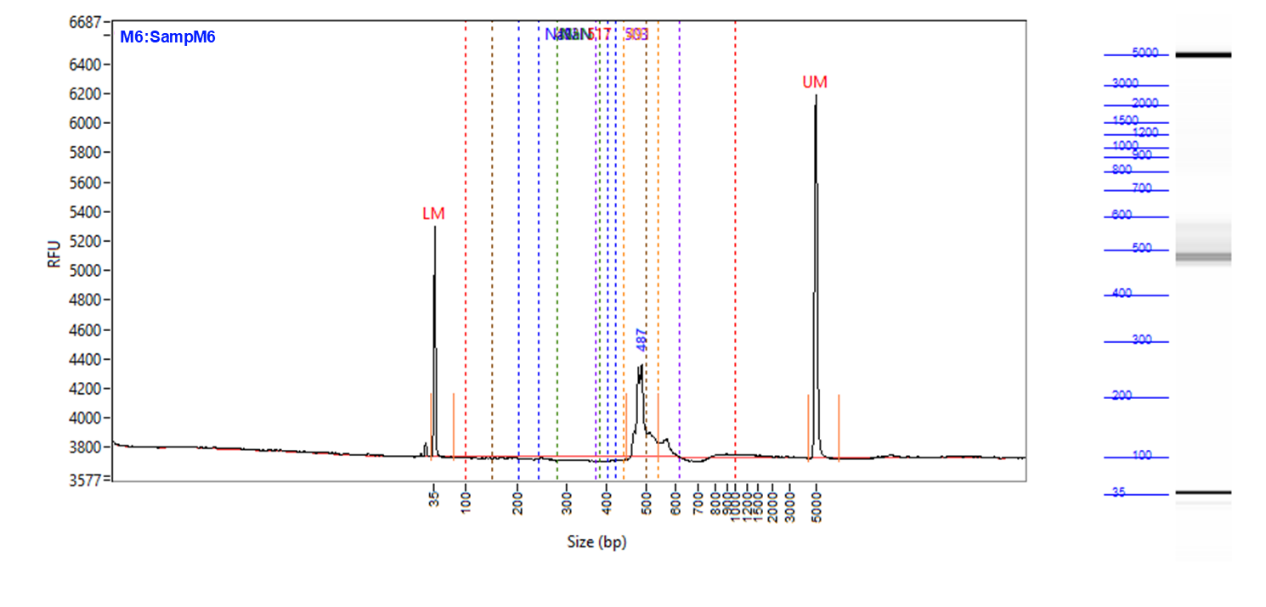
**

**m**

**
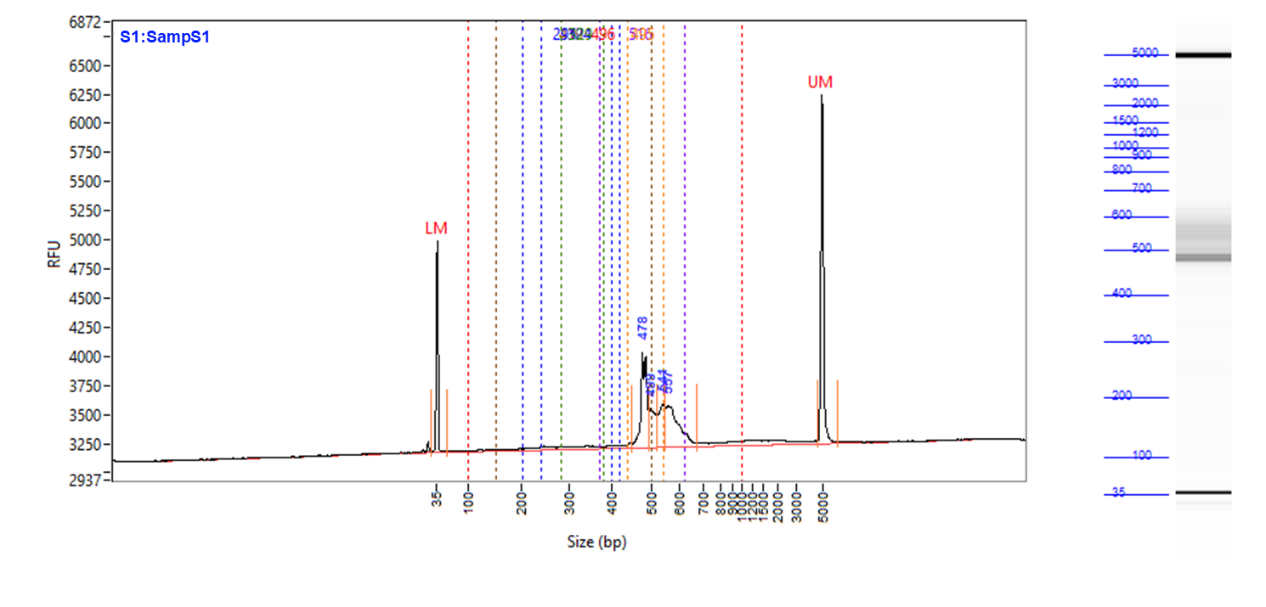
**

**n**

**
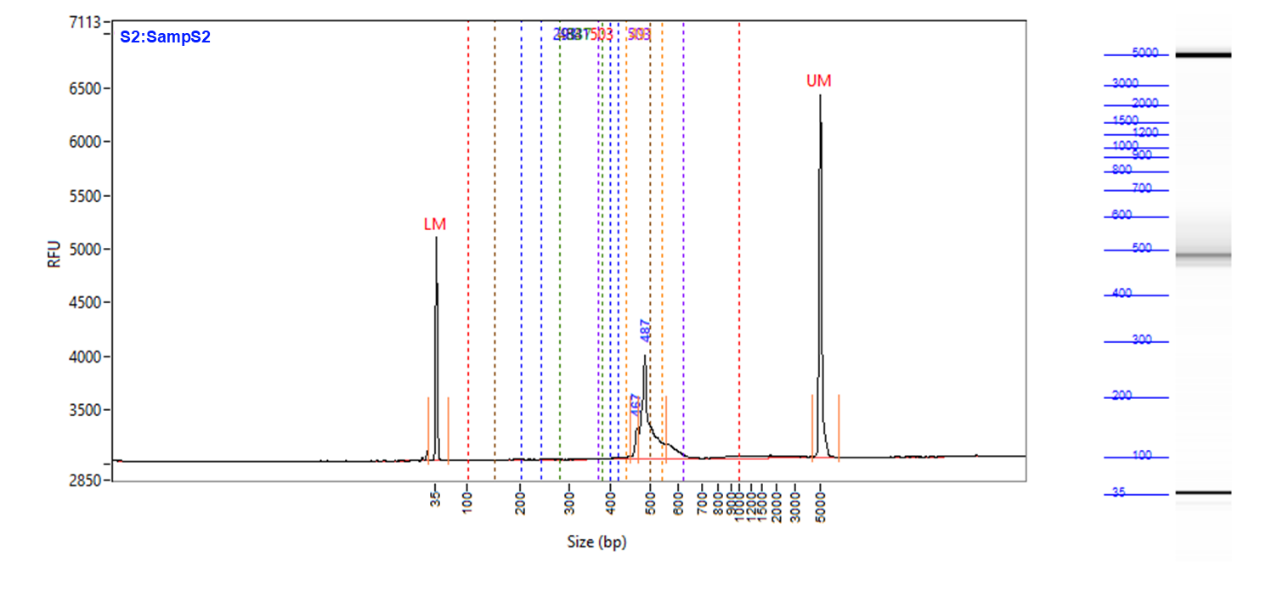
**

**o**

**
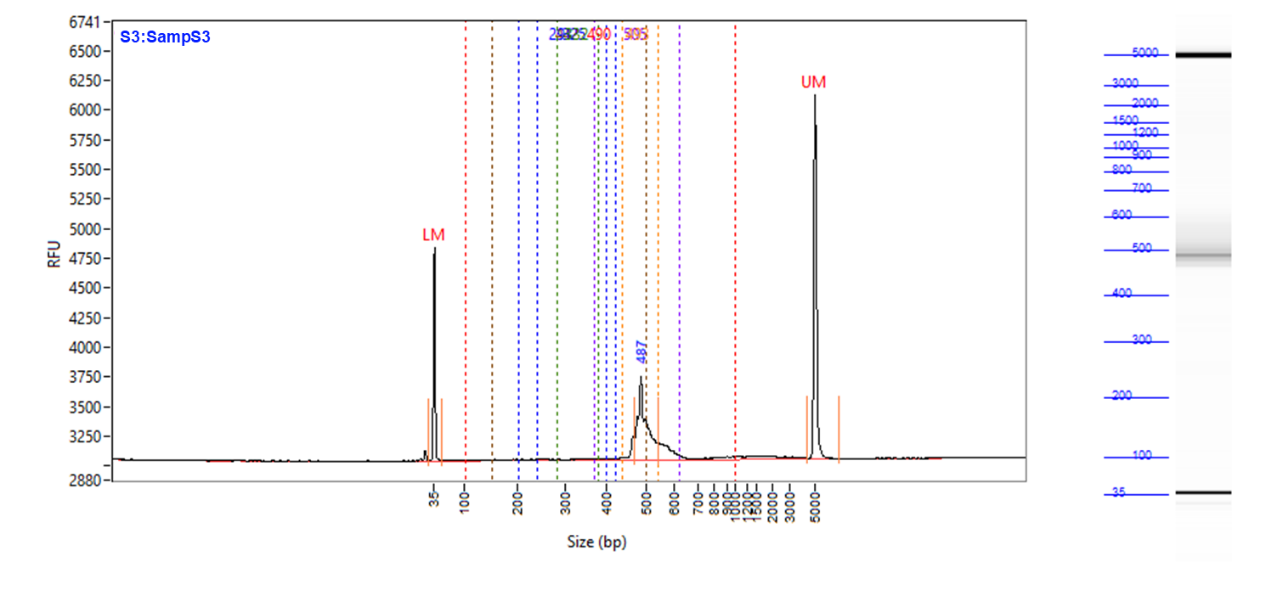
**

**p**

**
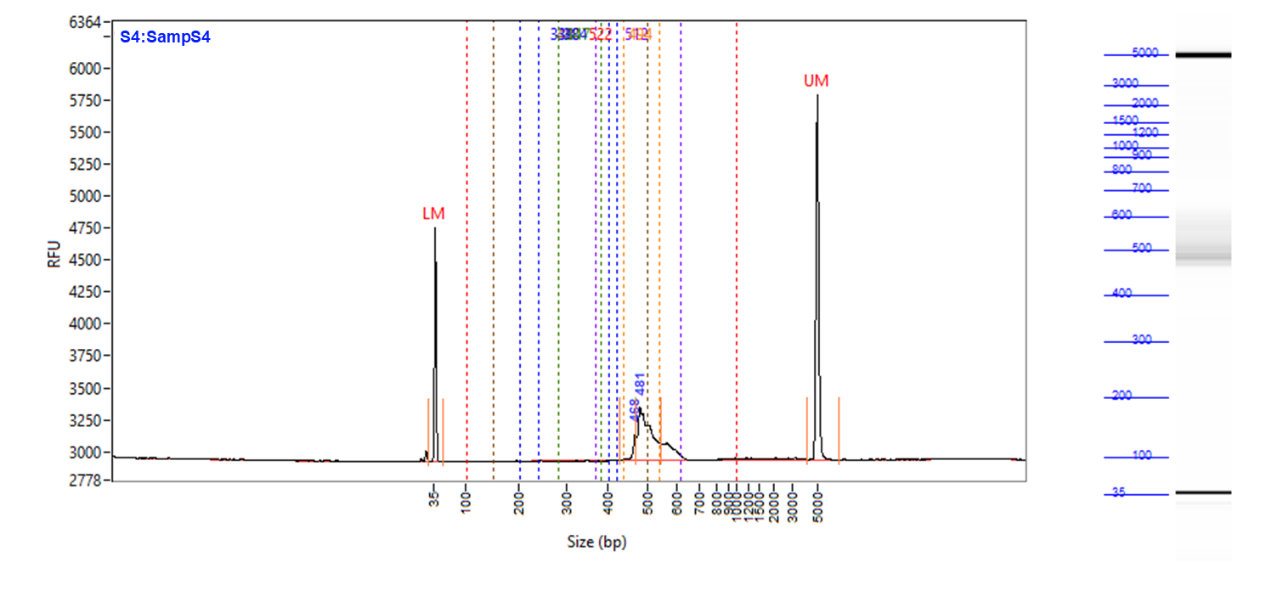
**

**q**

**
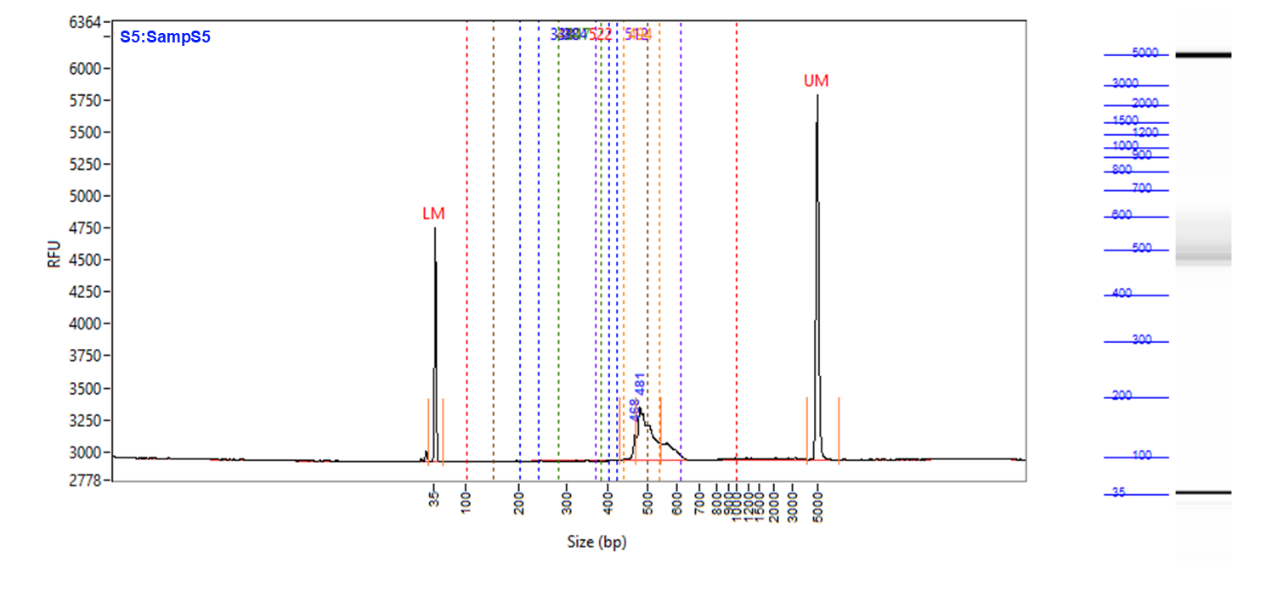
**

**r**

**
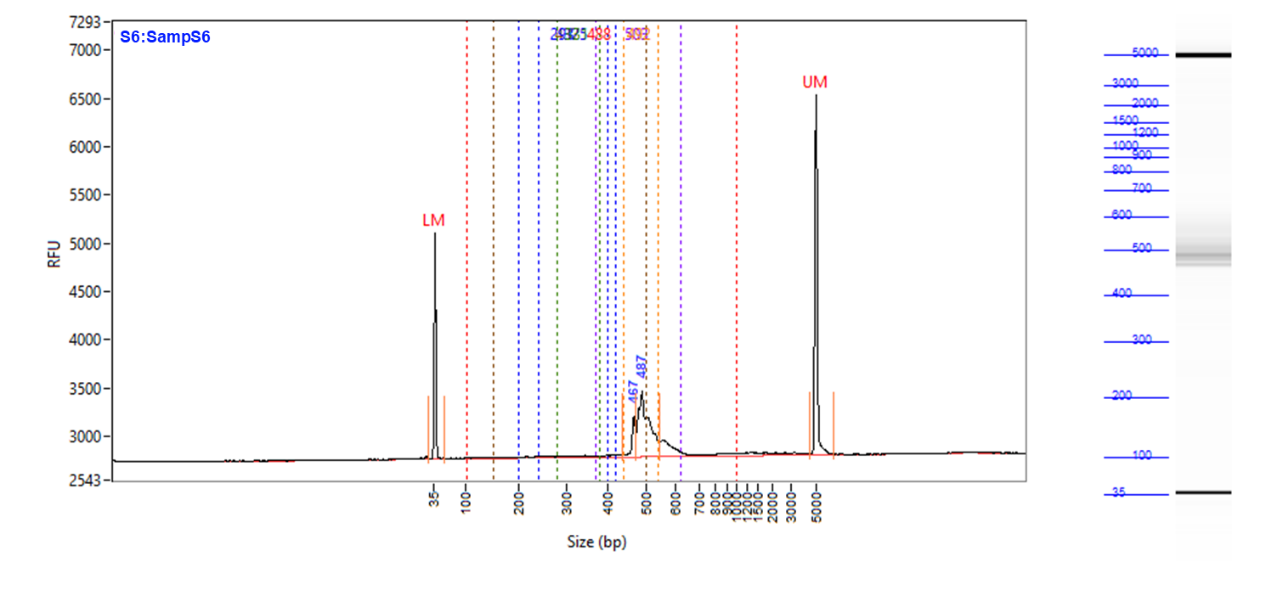
**

**Figure S2:** Analysis of peak-heights in agarose gel electropherogram.


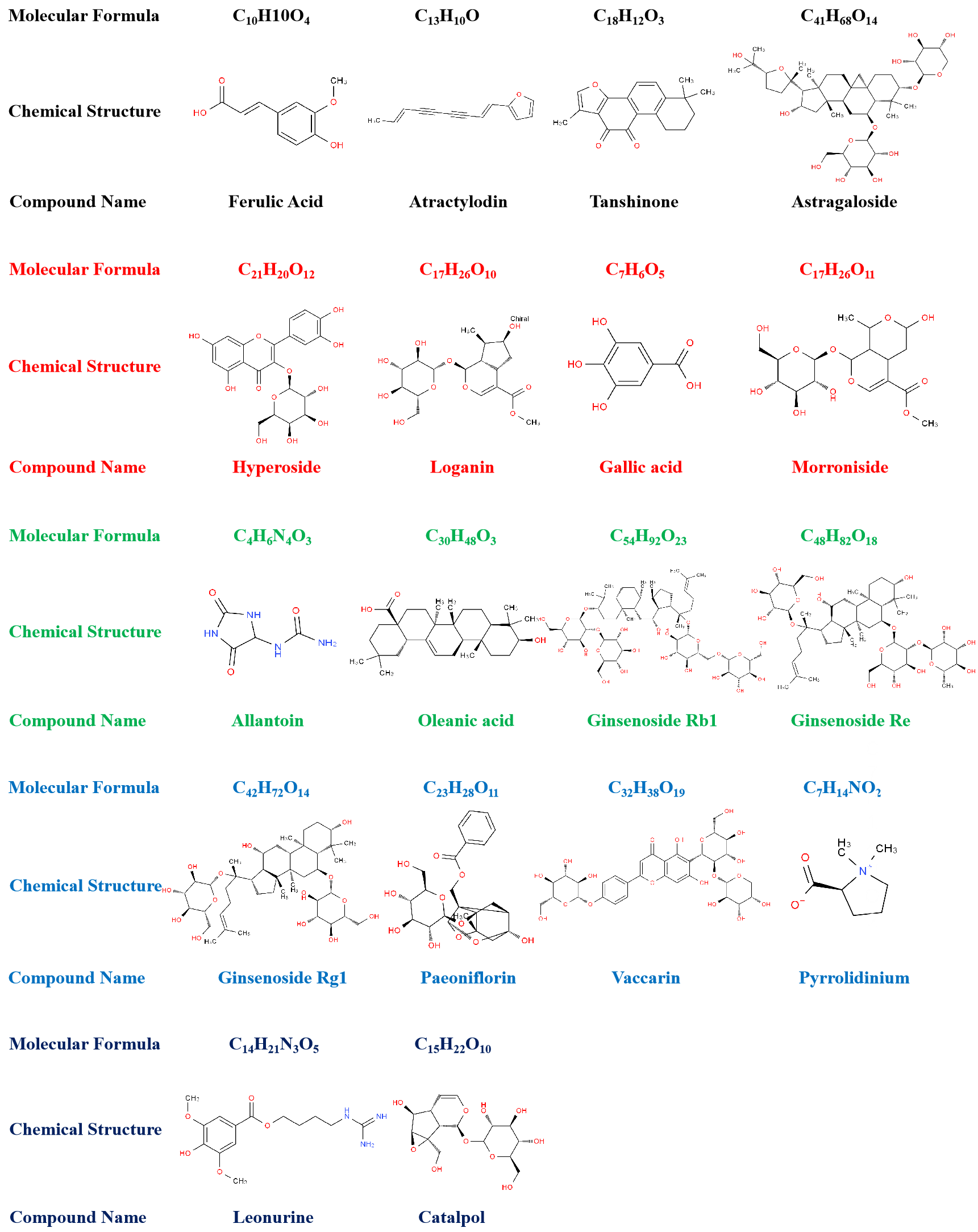


**FIGURE S3:** The molecular formulas and chemical structures of reference standards.

**a**


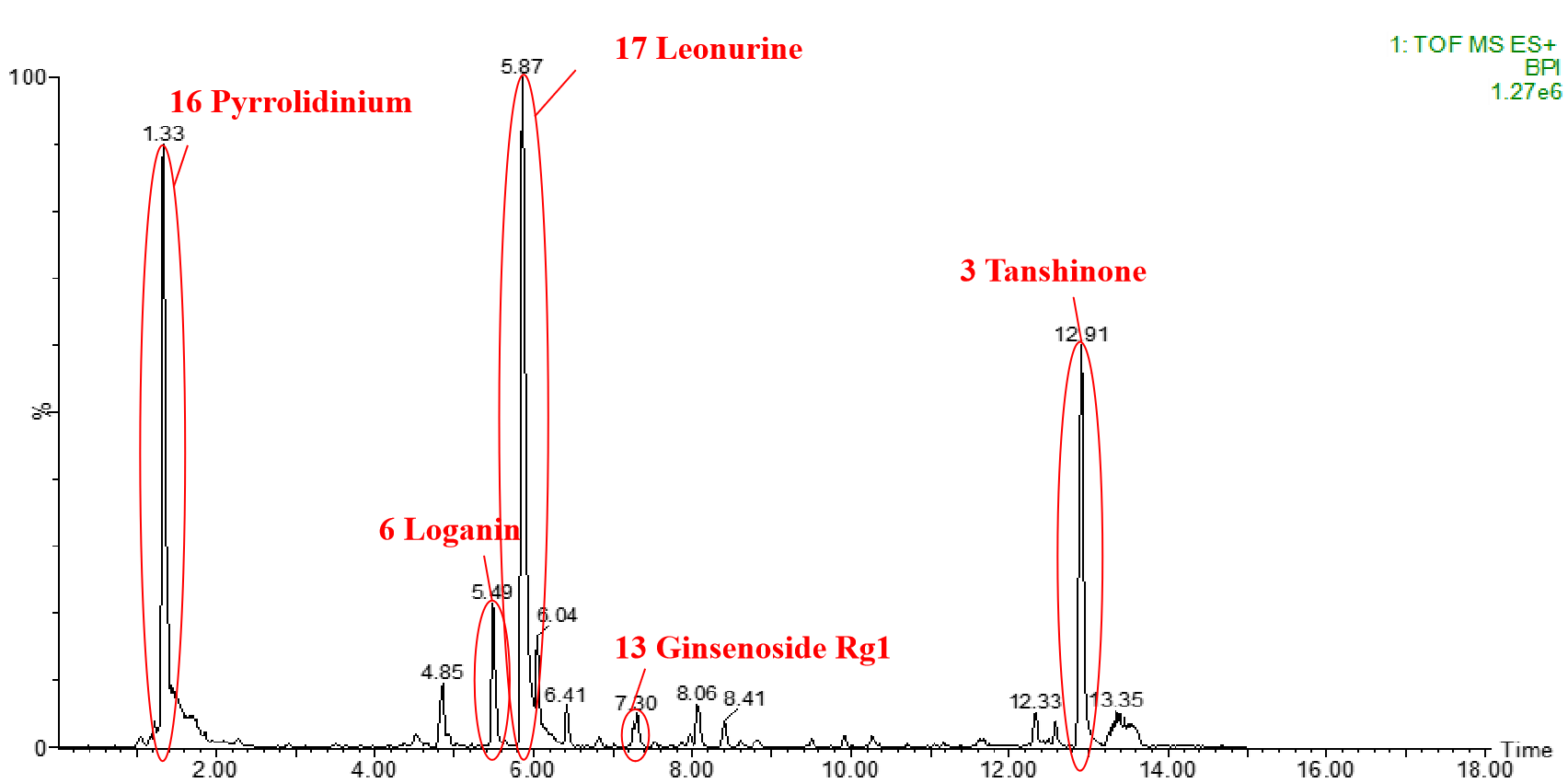


**b**


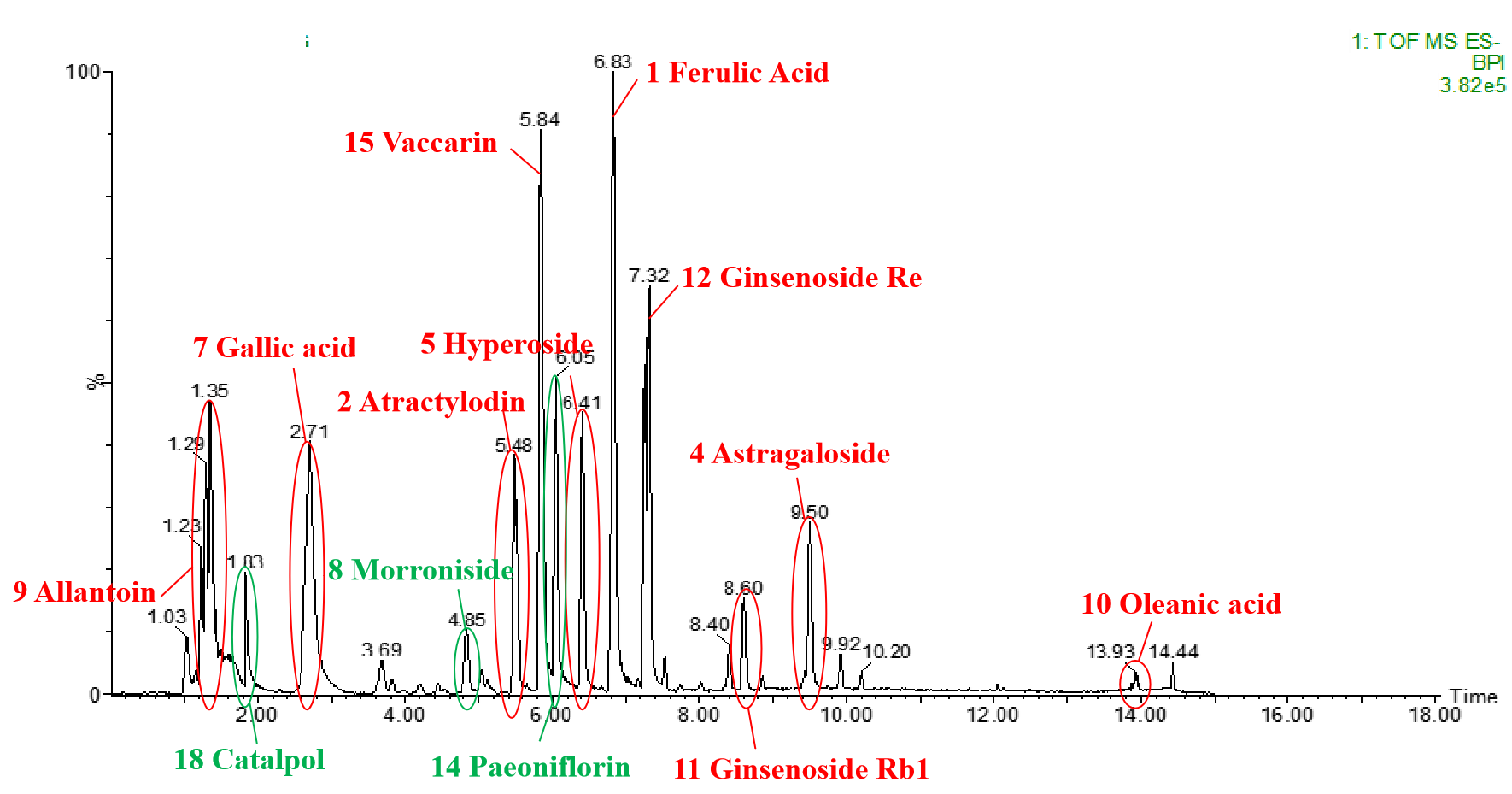


**c**

**
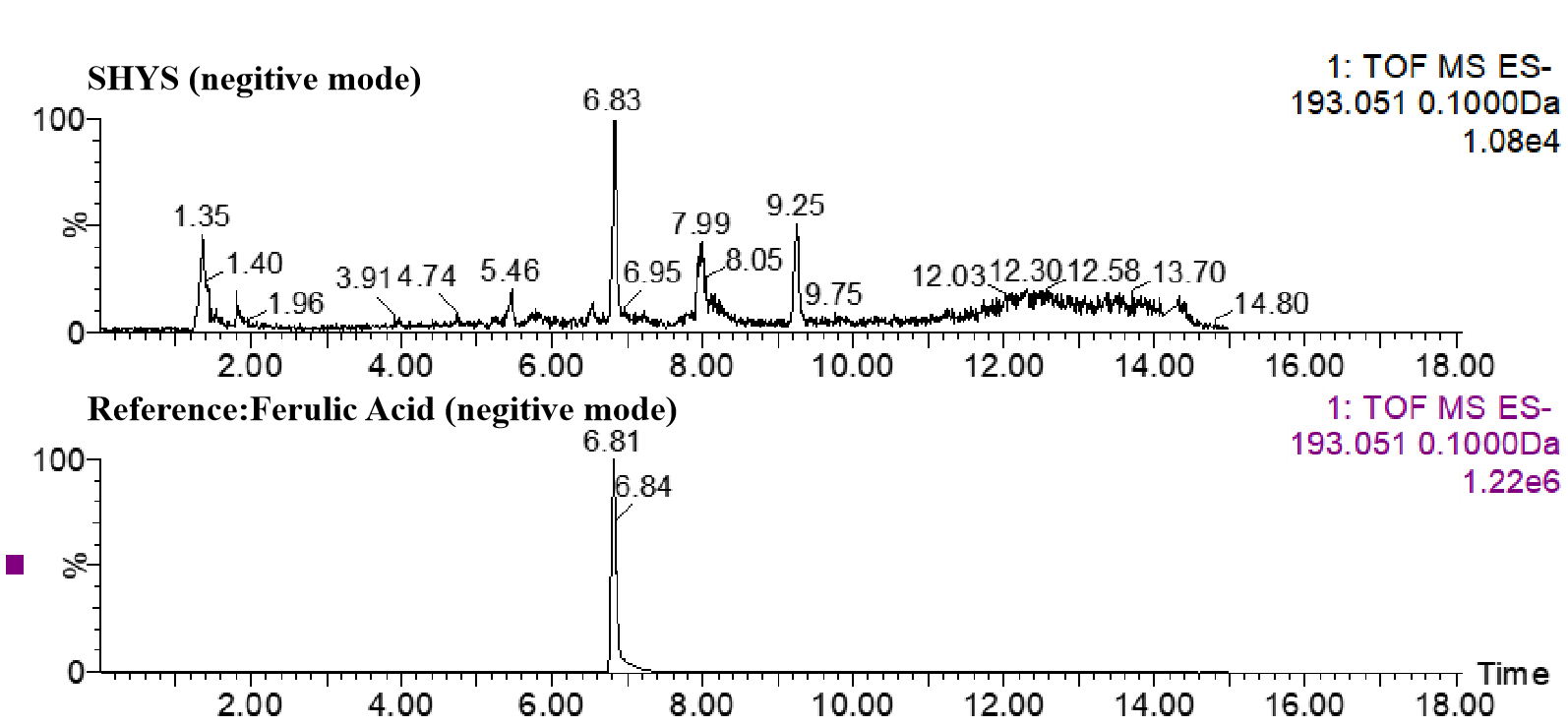
**

**d**

**
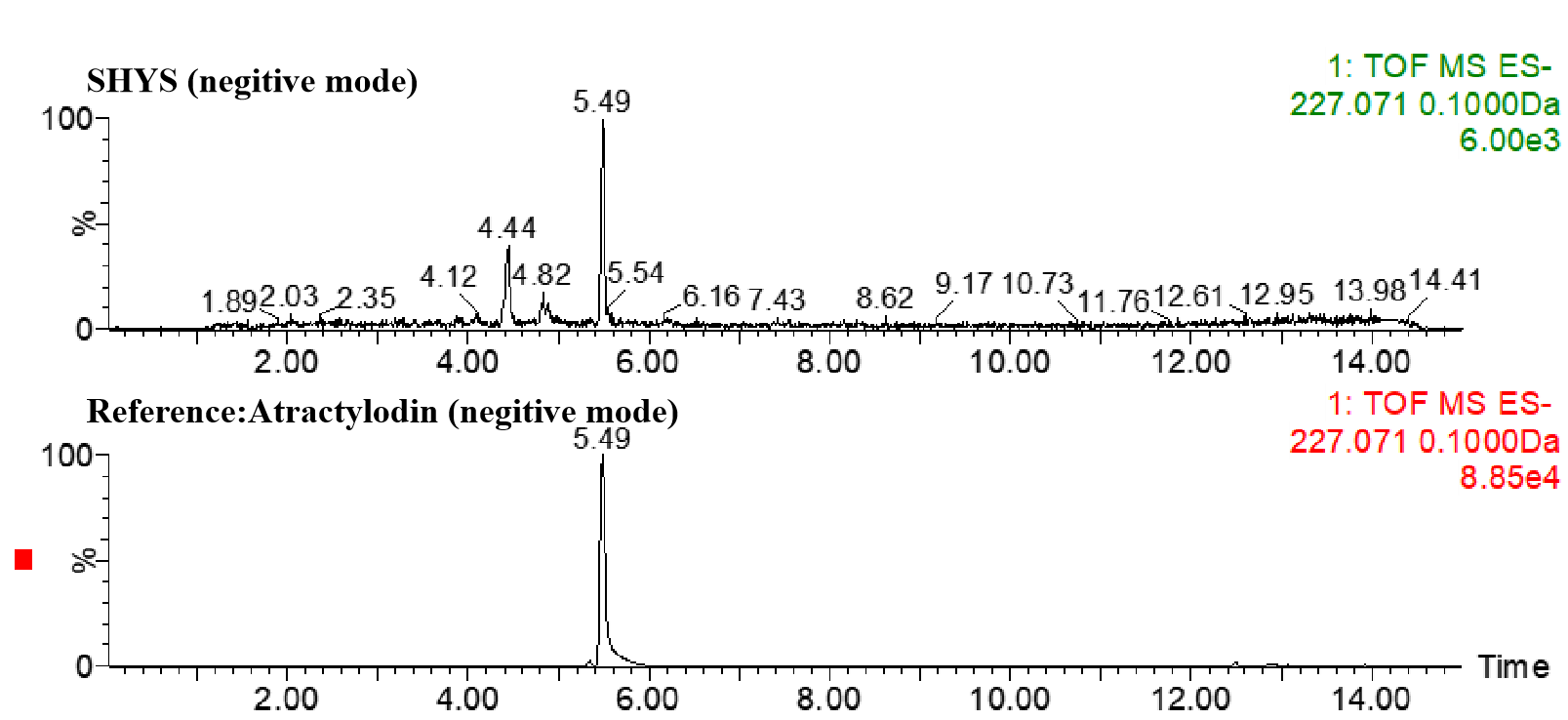
**

**e**

**
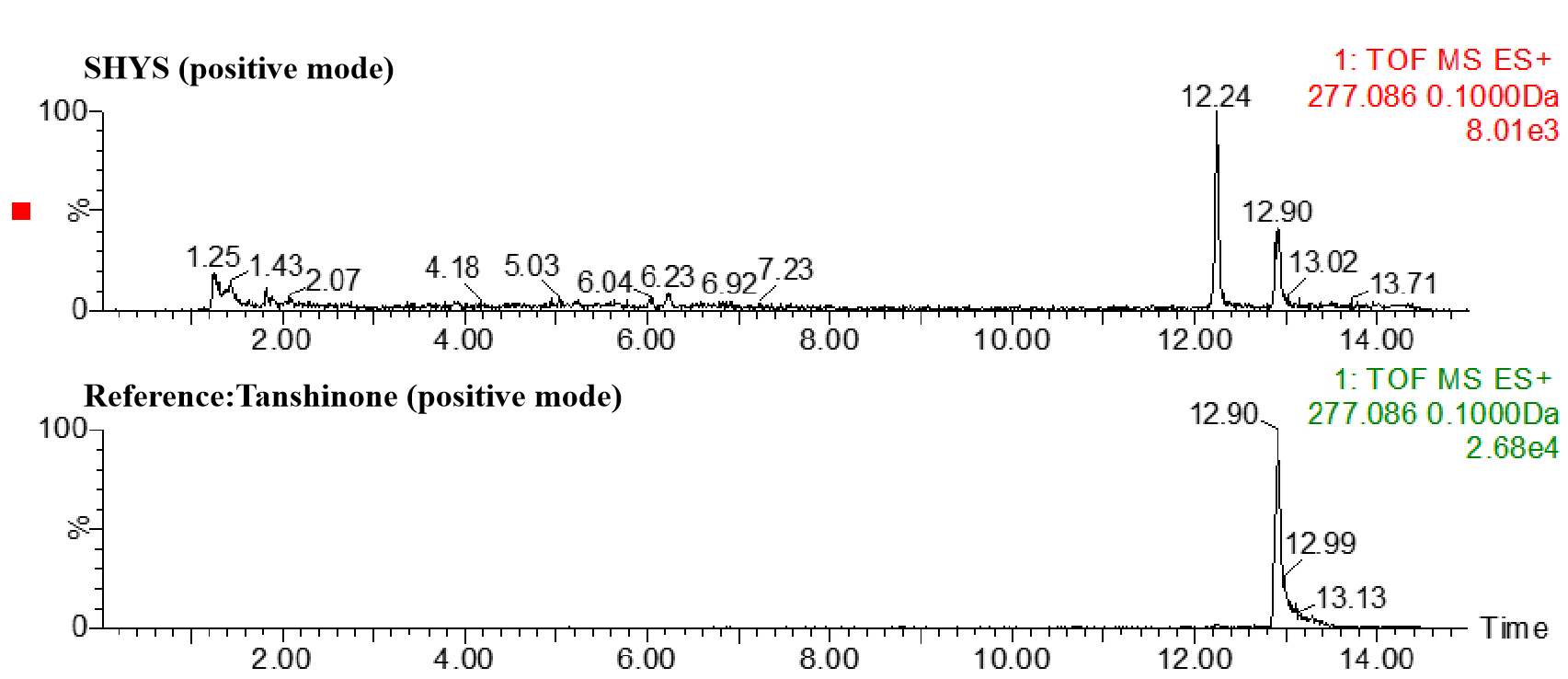
**

**f**

**
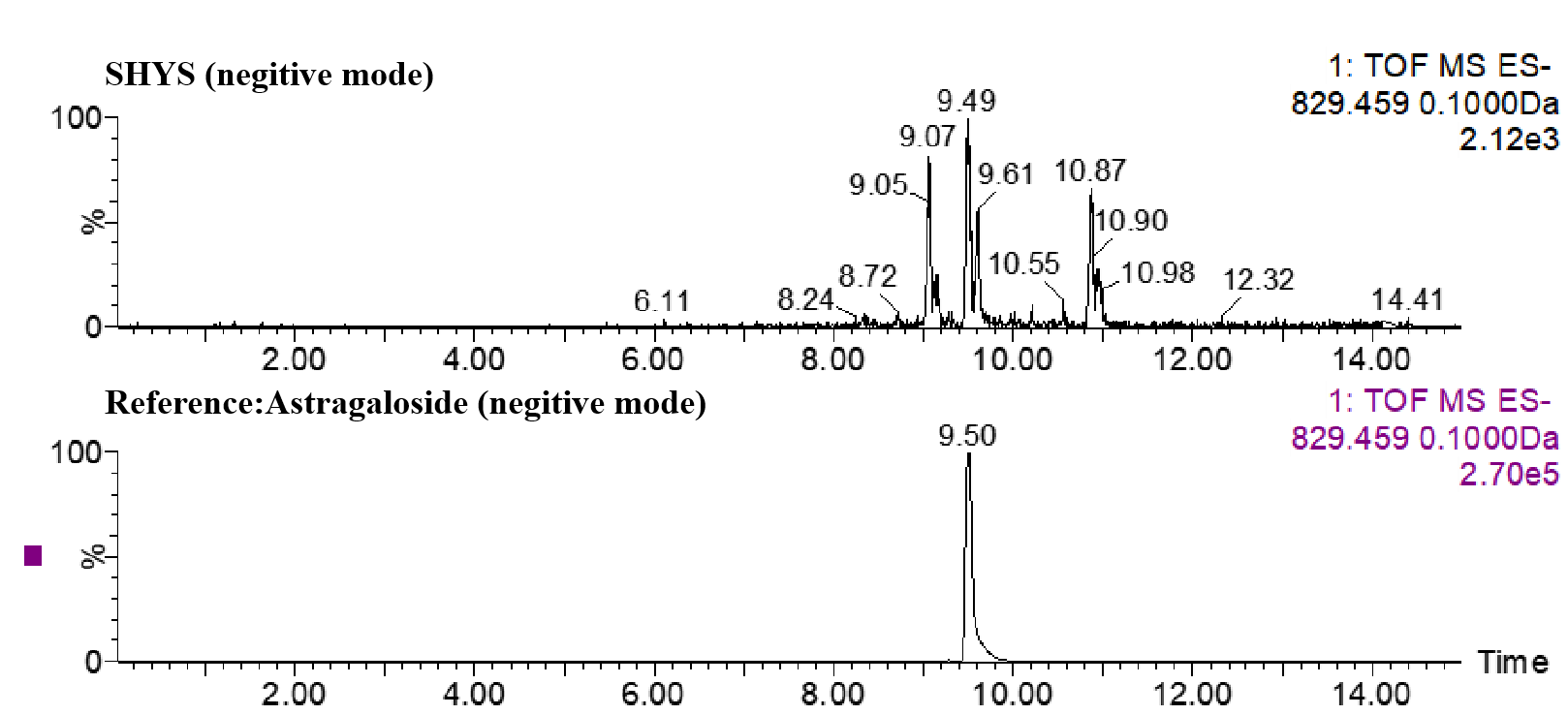
**

**g**

**
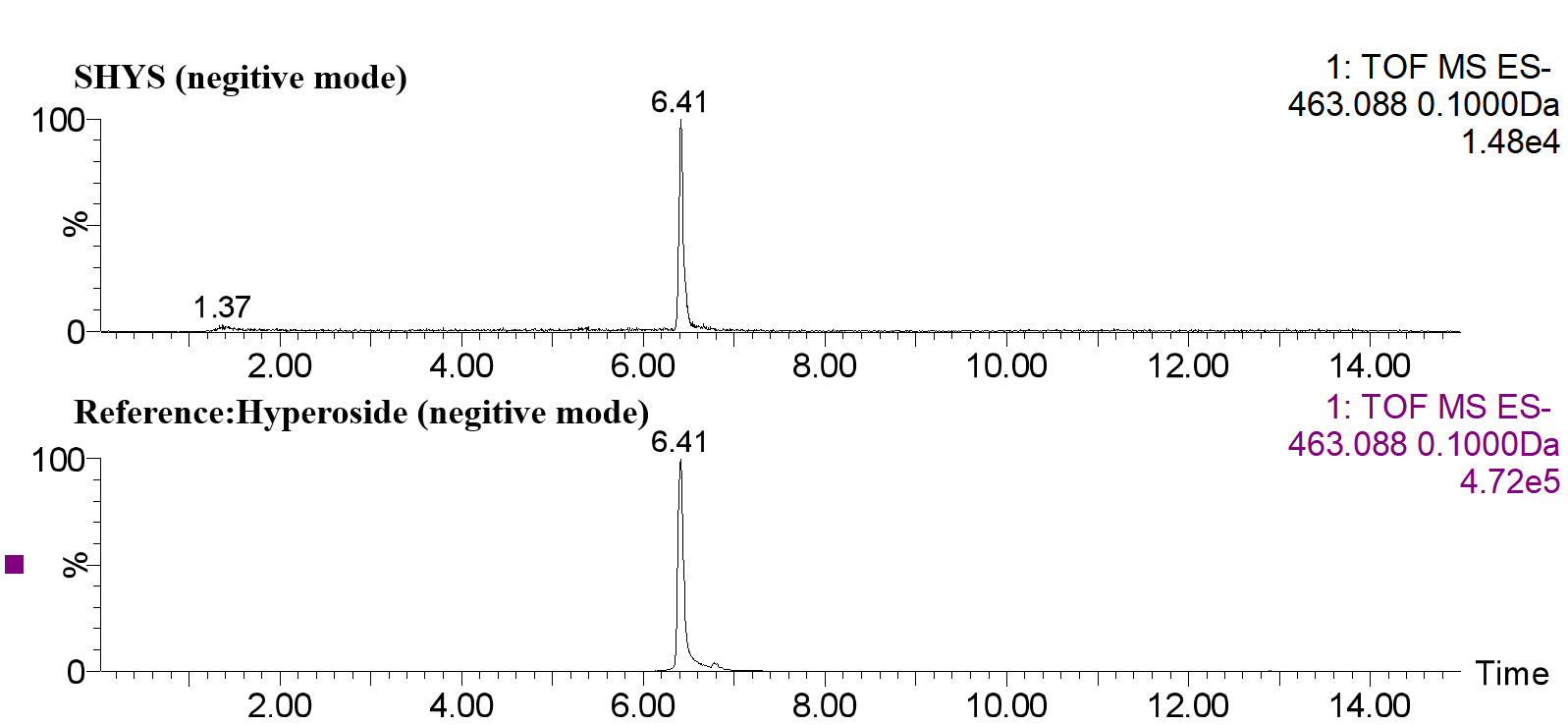
**

**h**

**
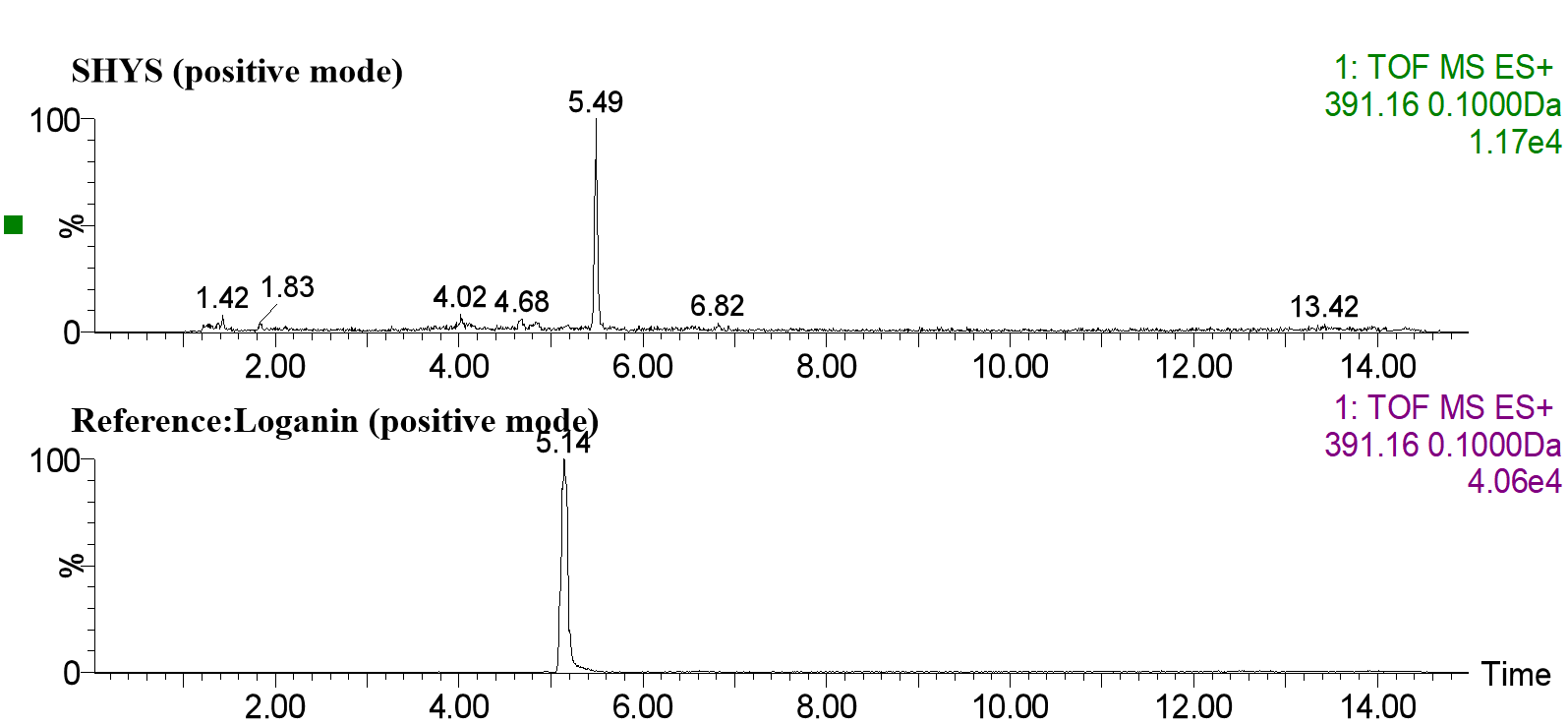
**

**i**

**
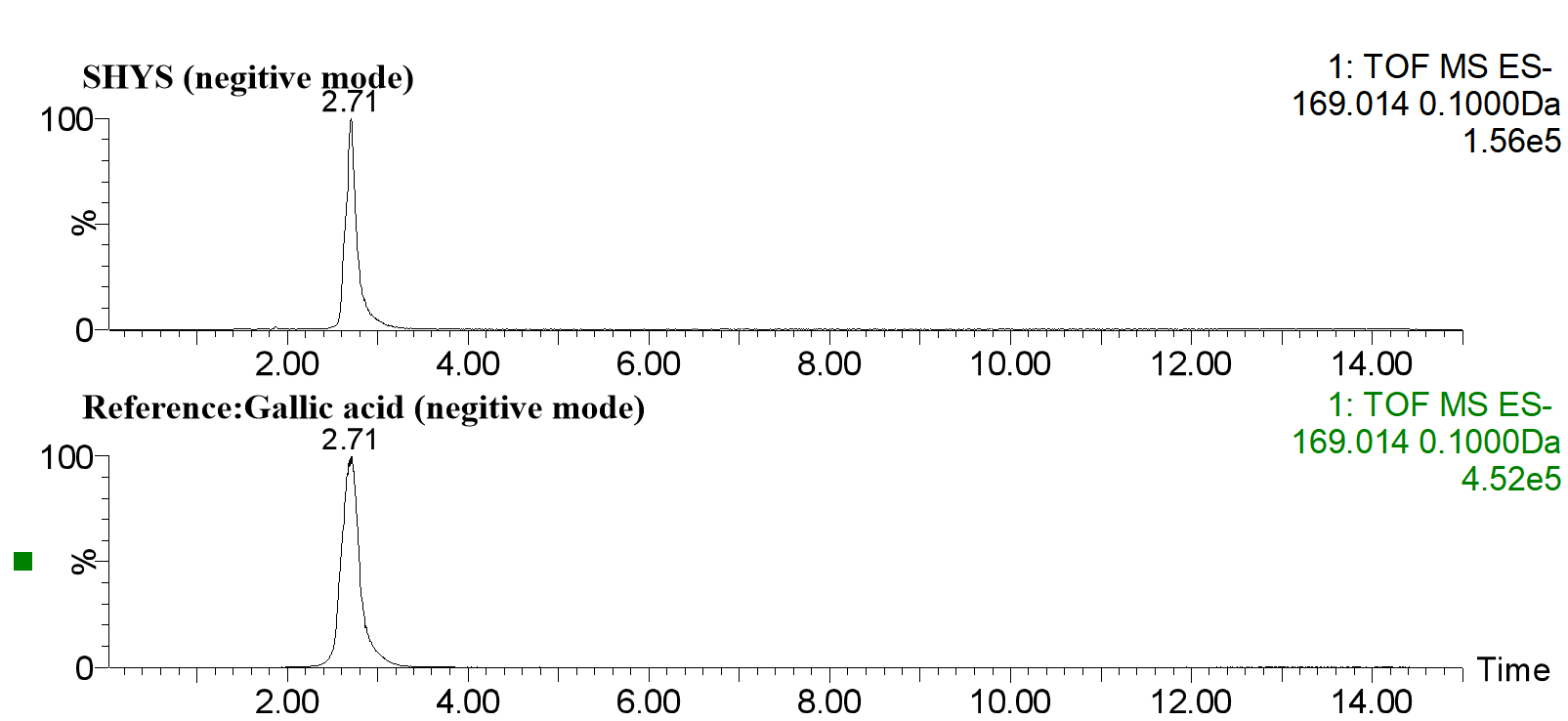
**

**j**

**
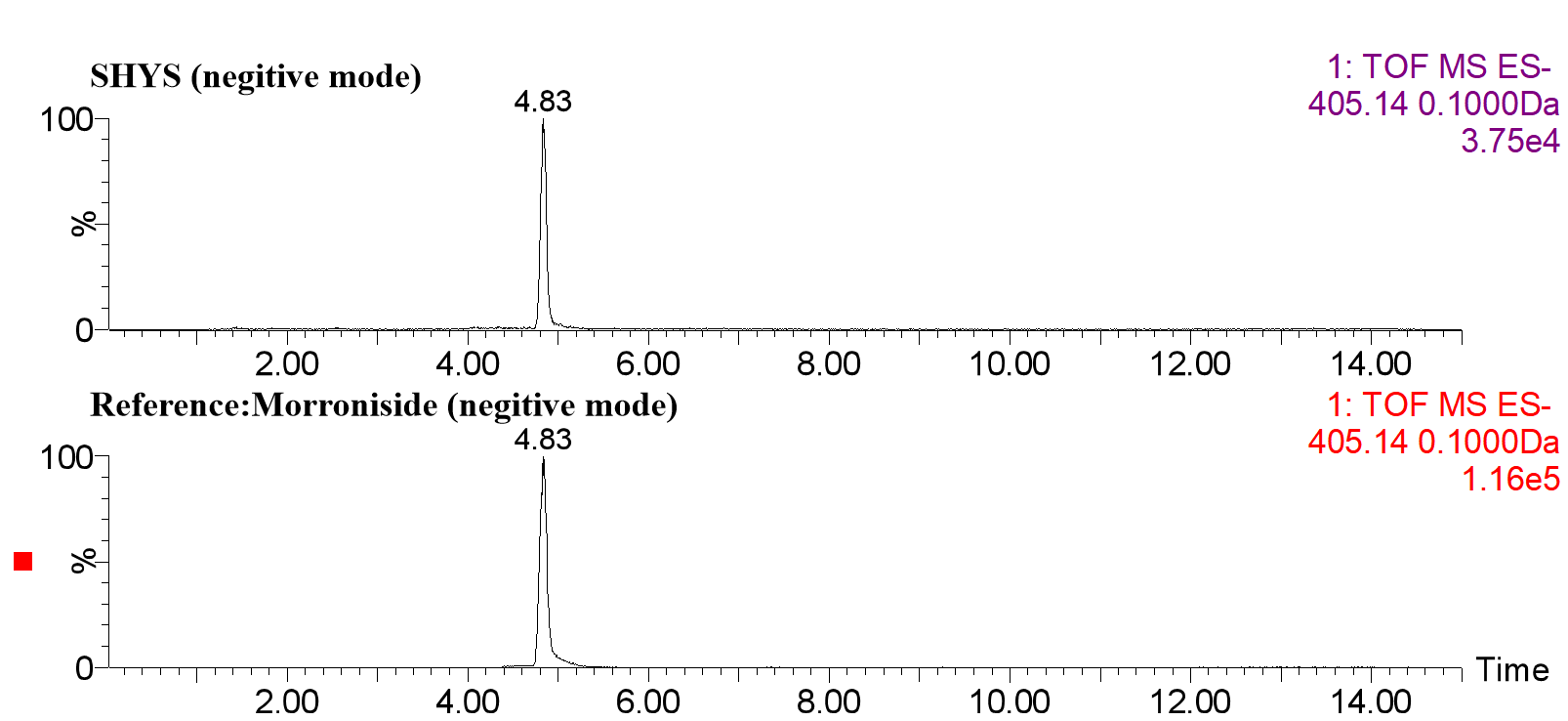
**

**k**

**
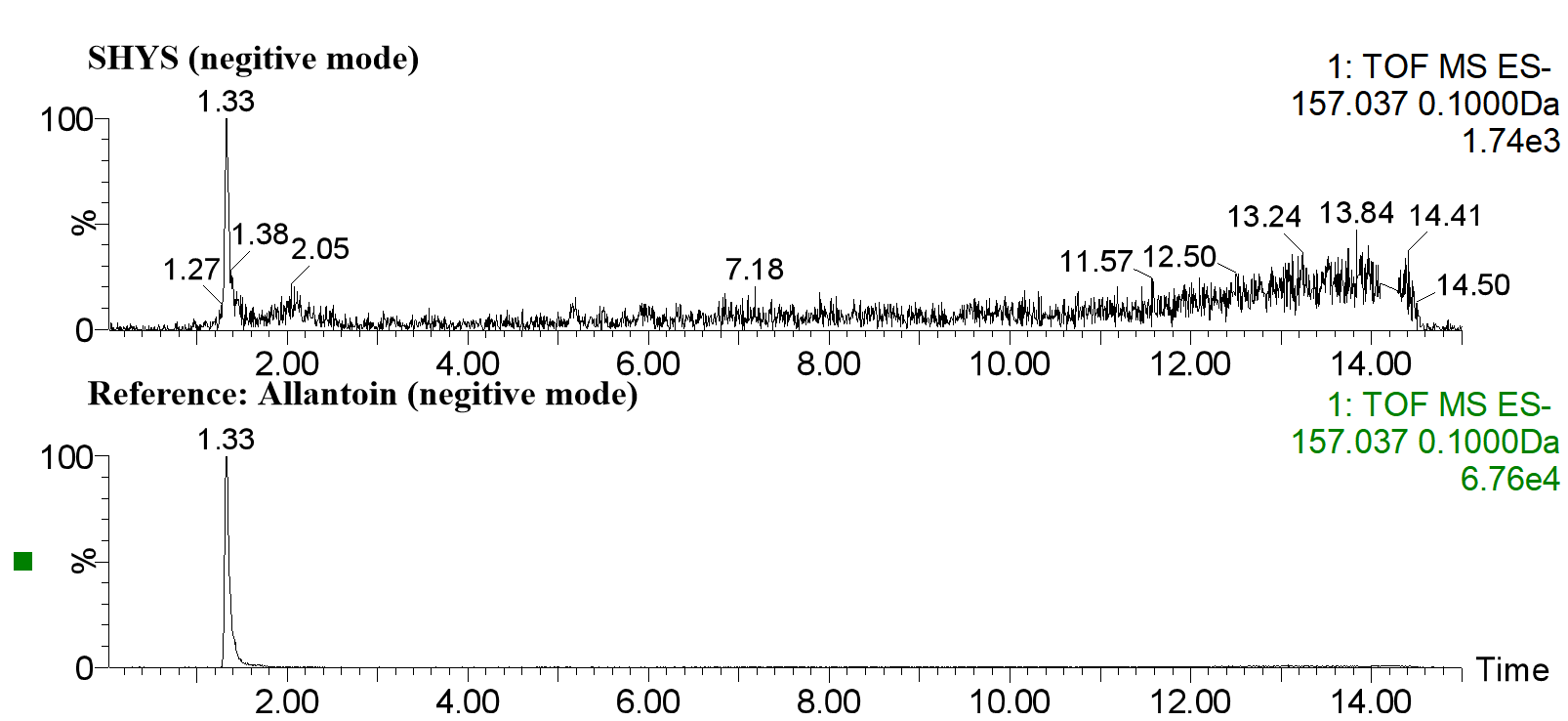
**

**l**

**
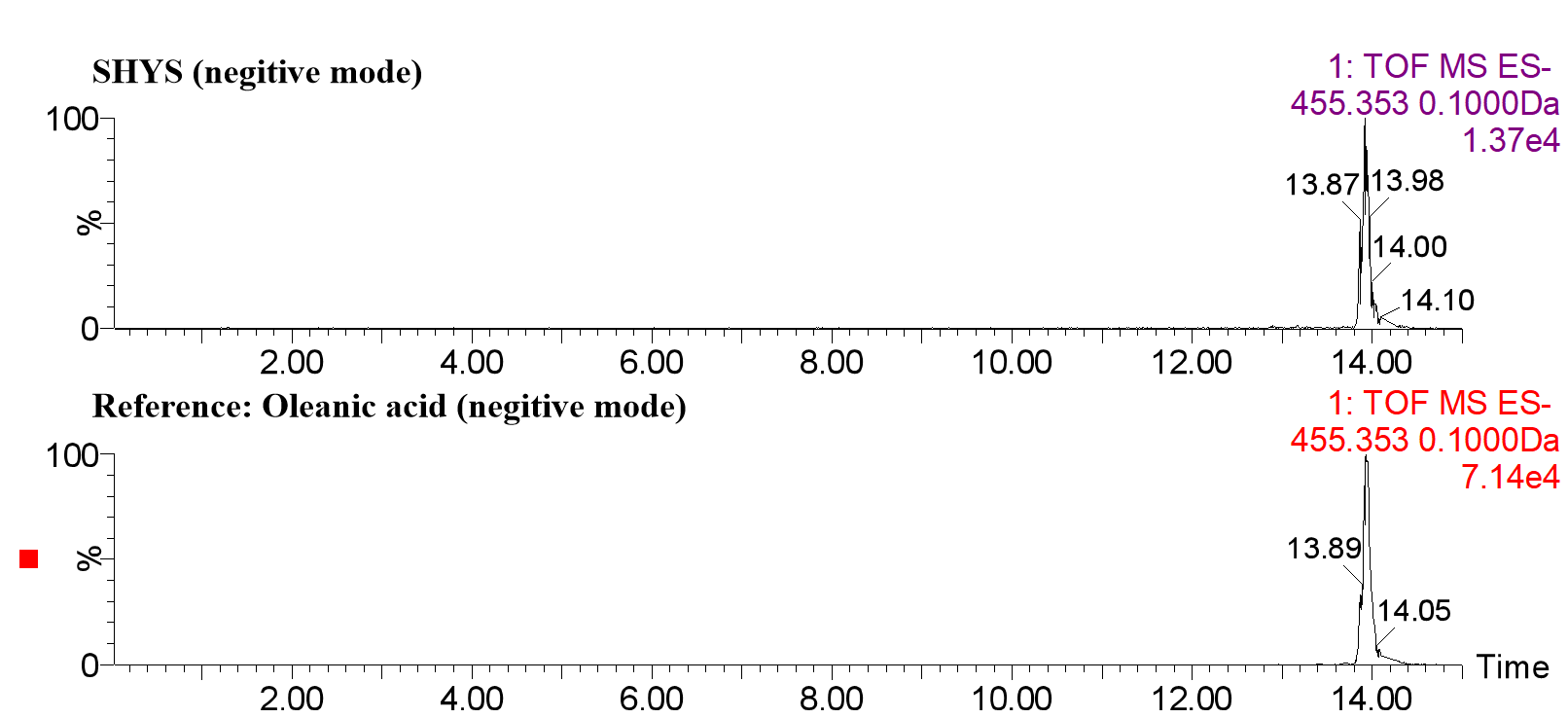
**

**m**

**
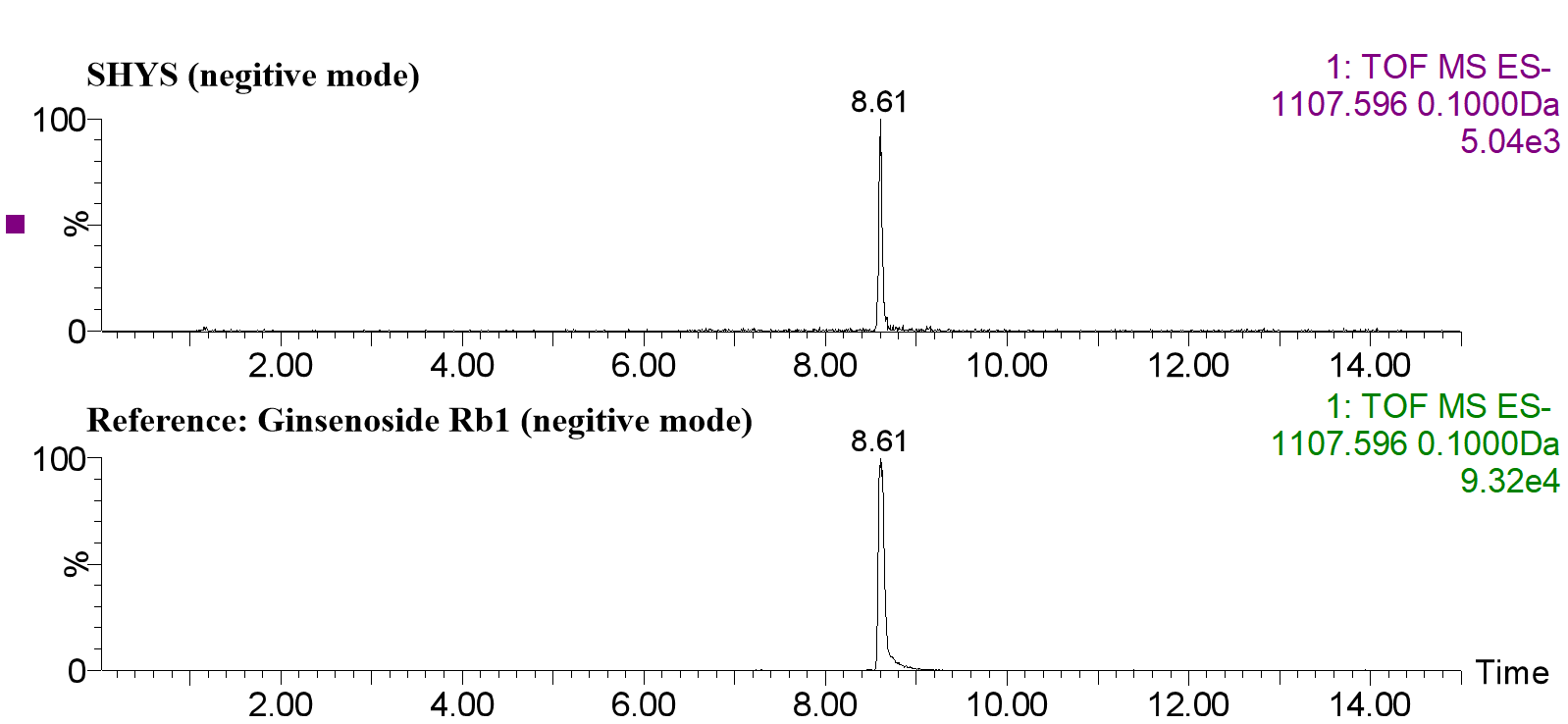
**

**n**

**
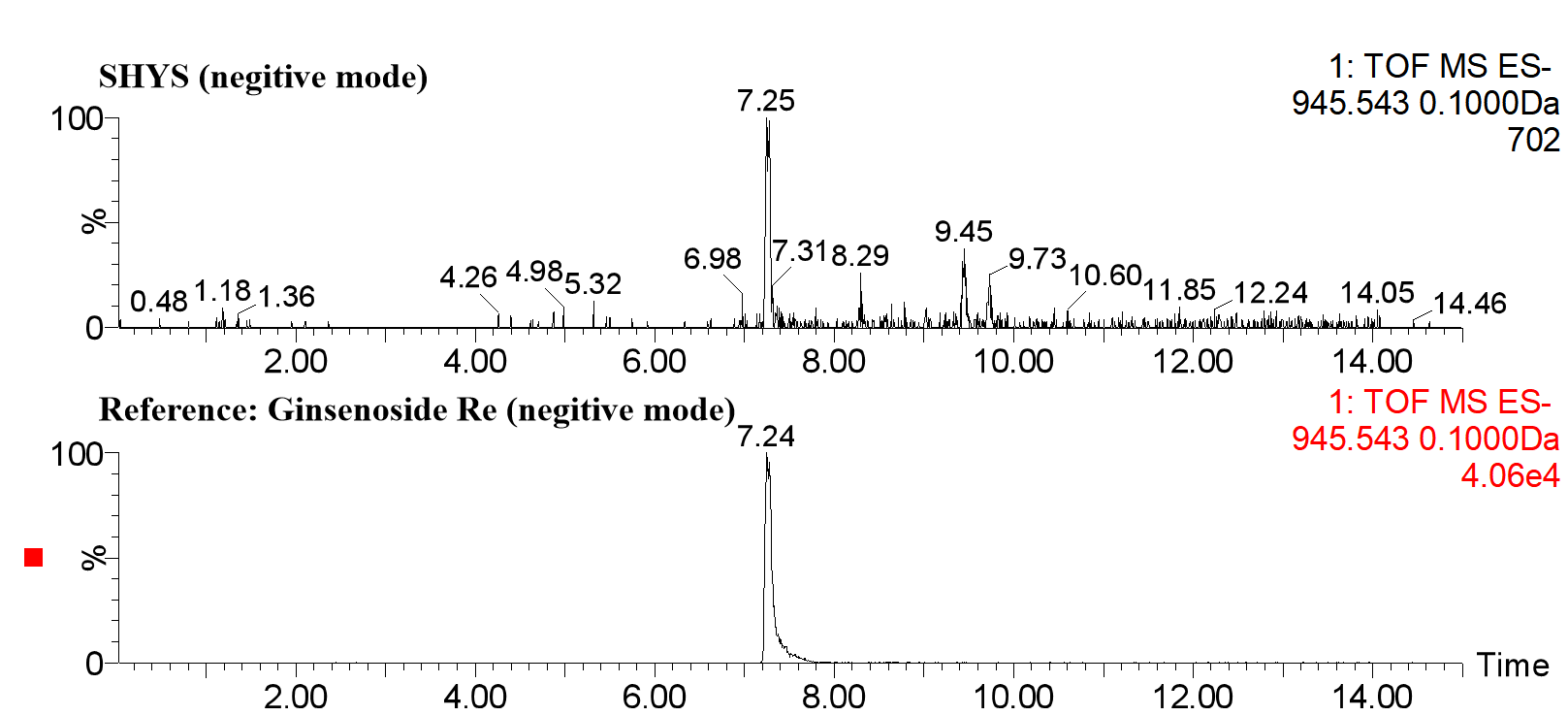
**

**o**

**
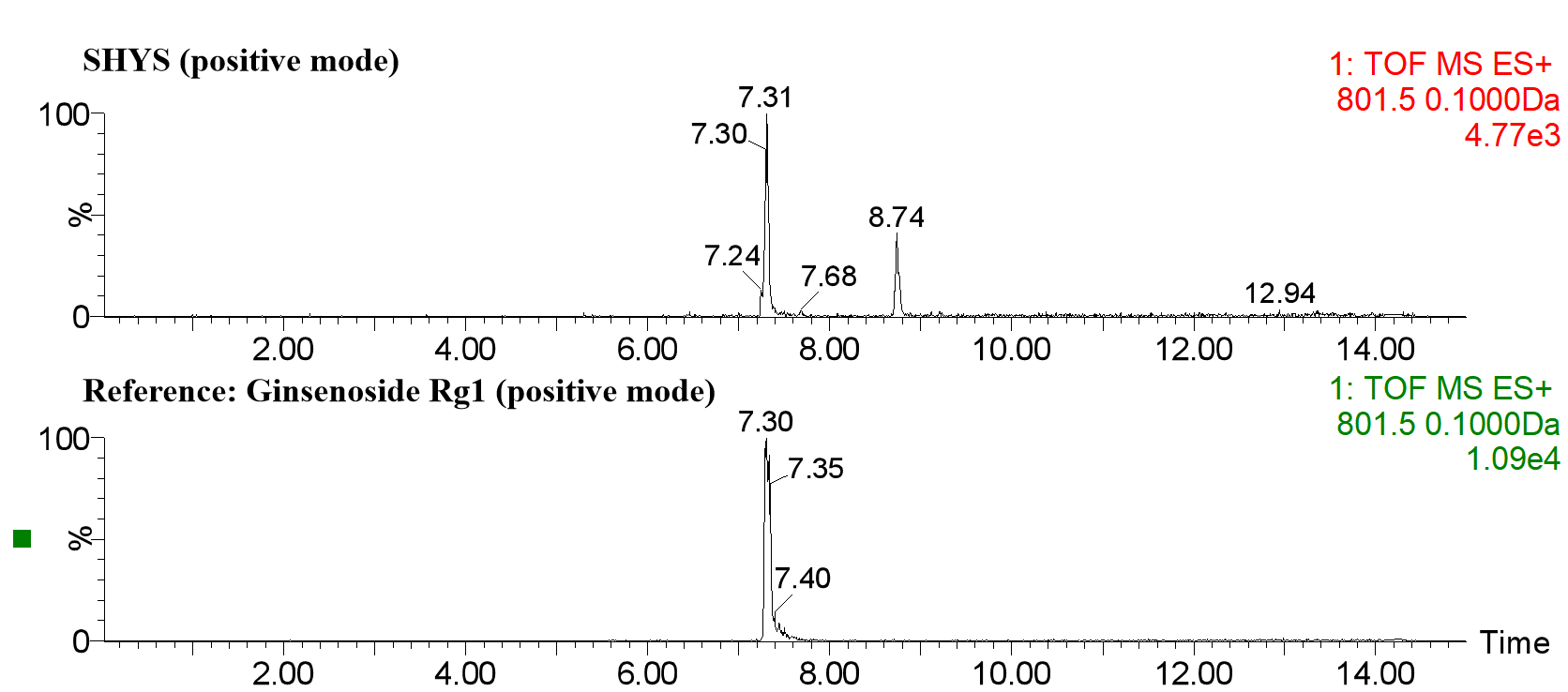
**

**p**

**
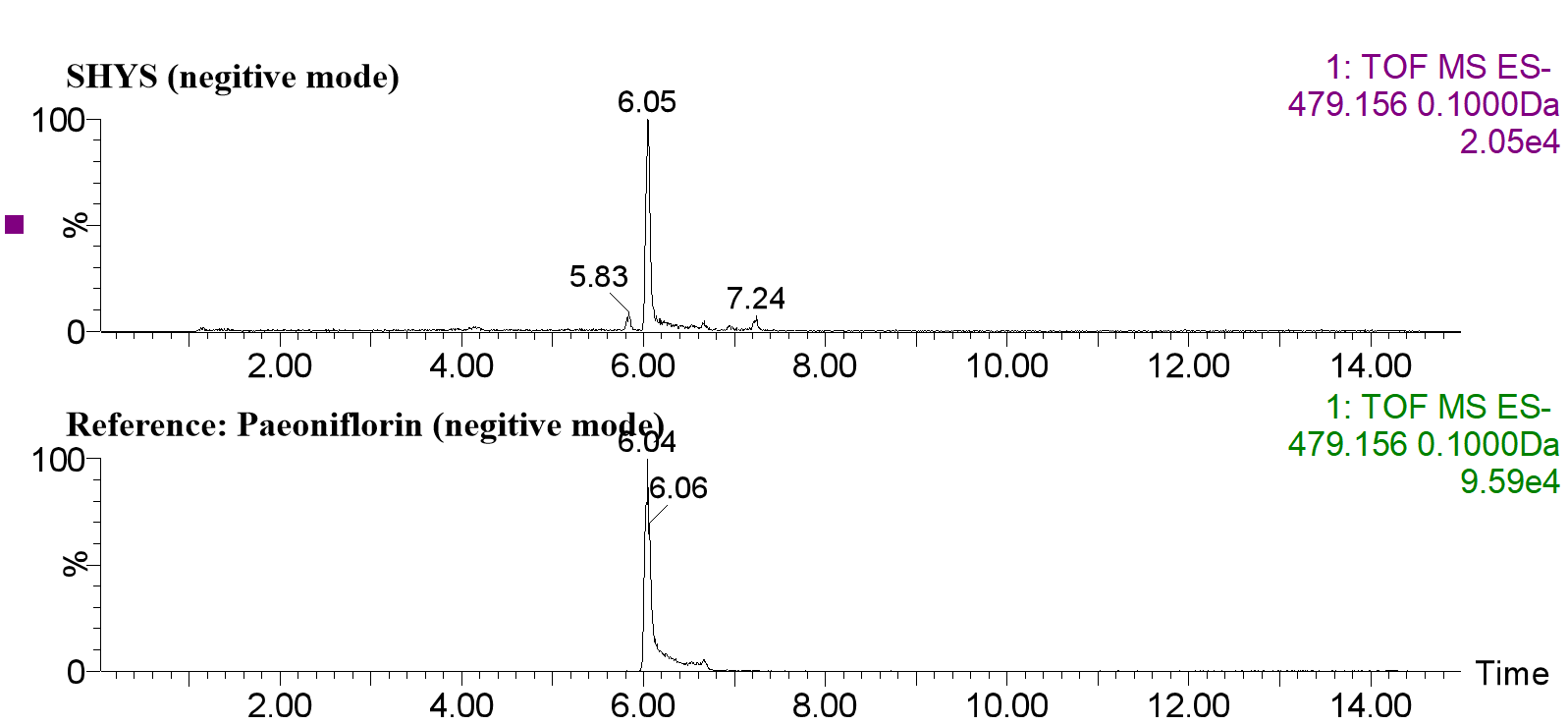
**

**q**

**
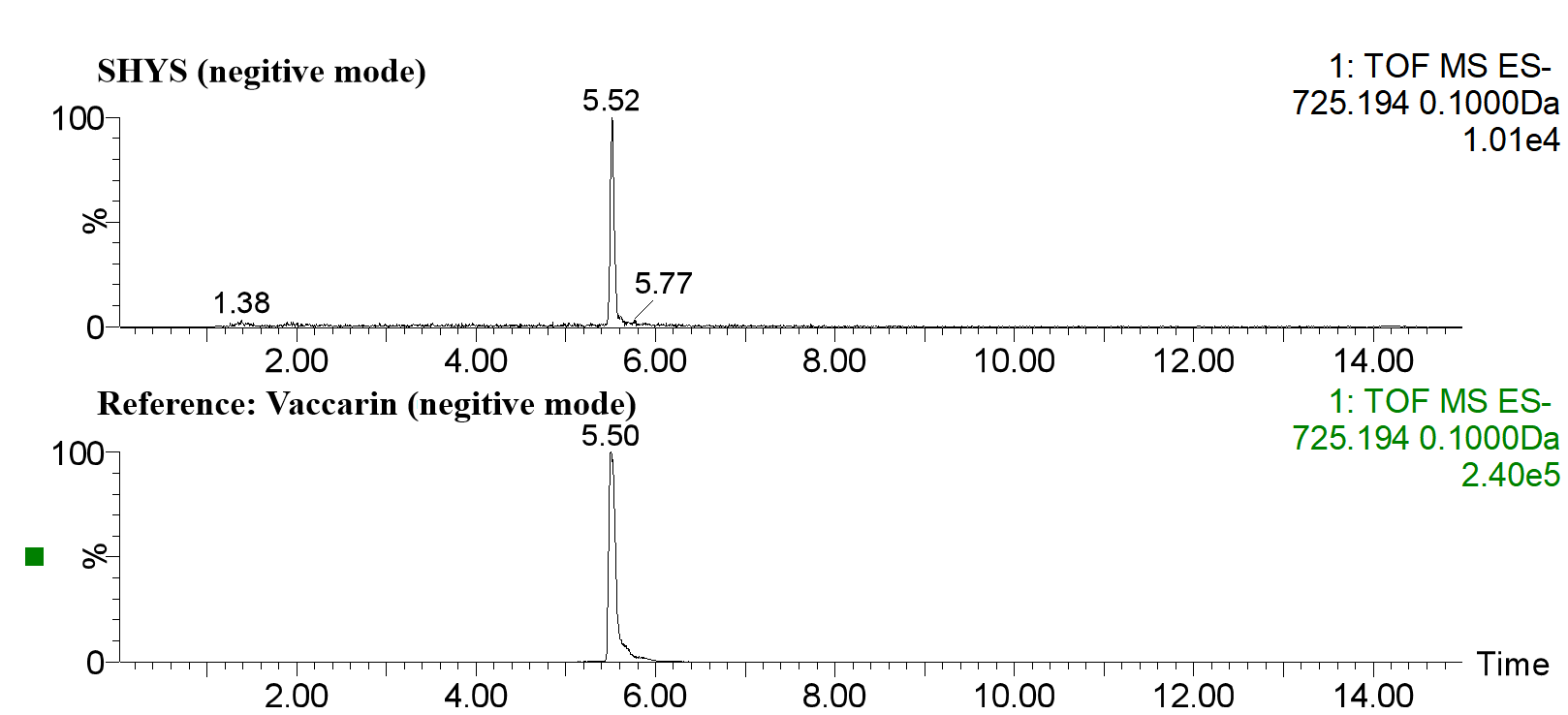
**

**r**

**
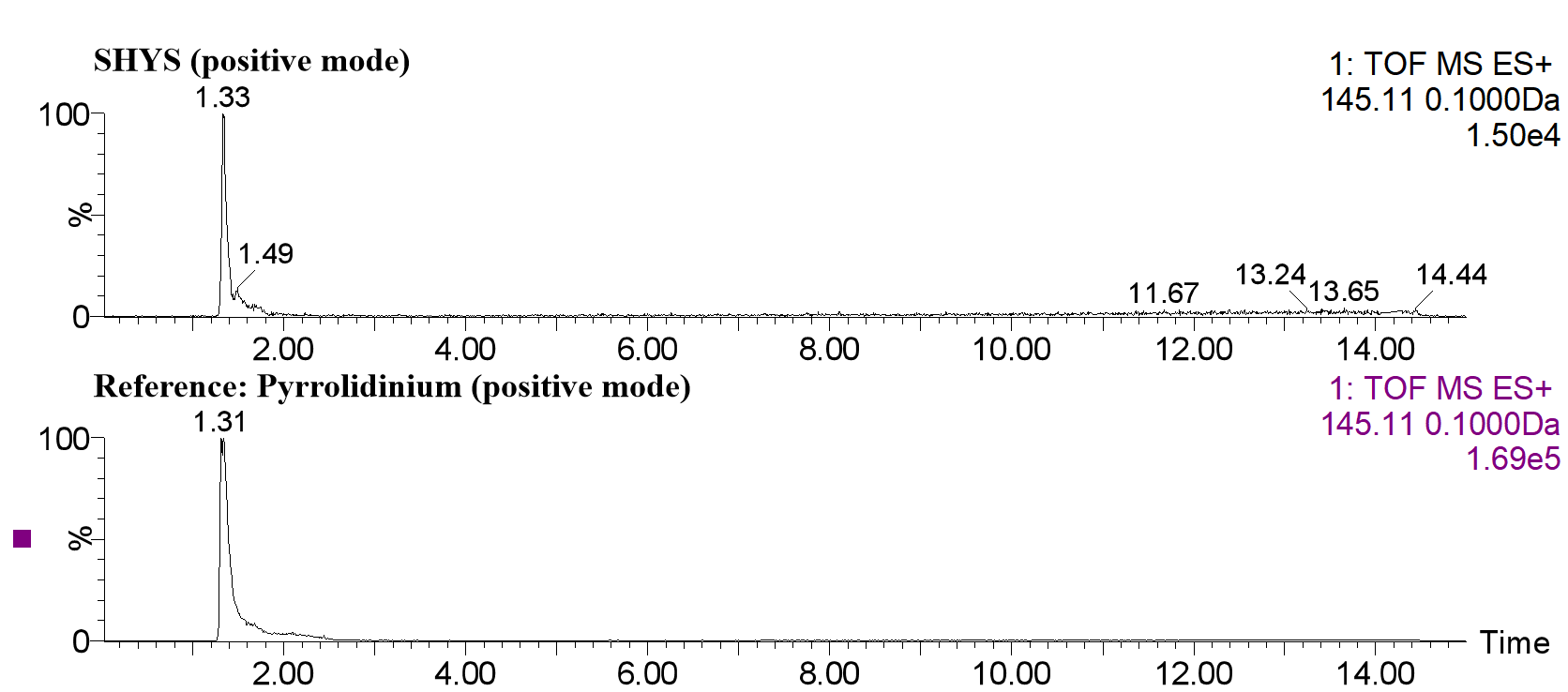
**

**s**

**
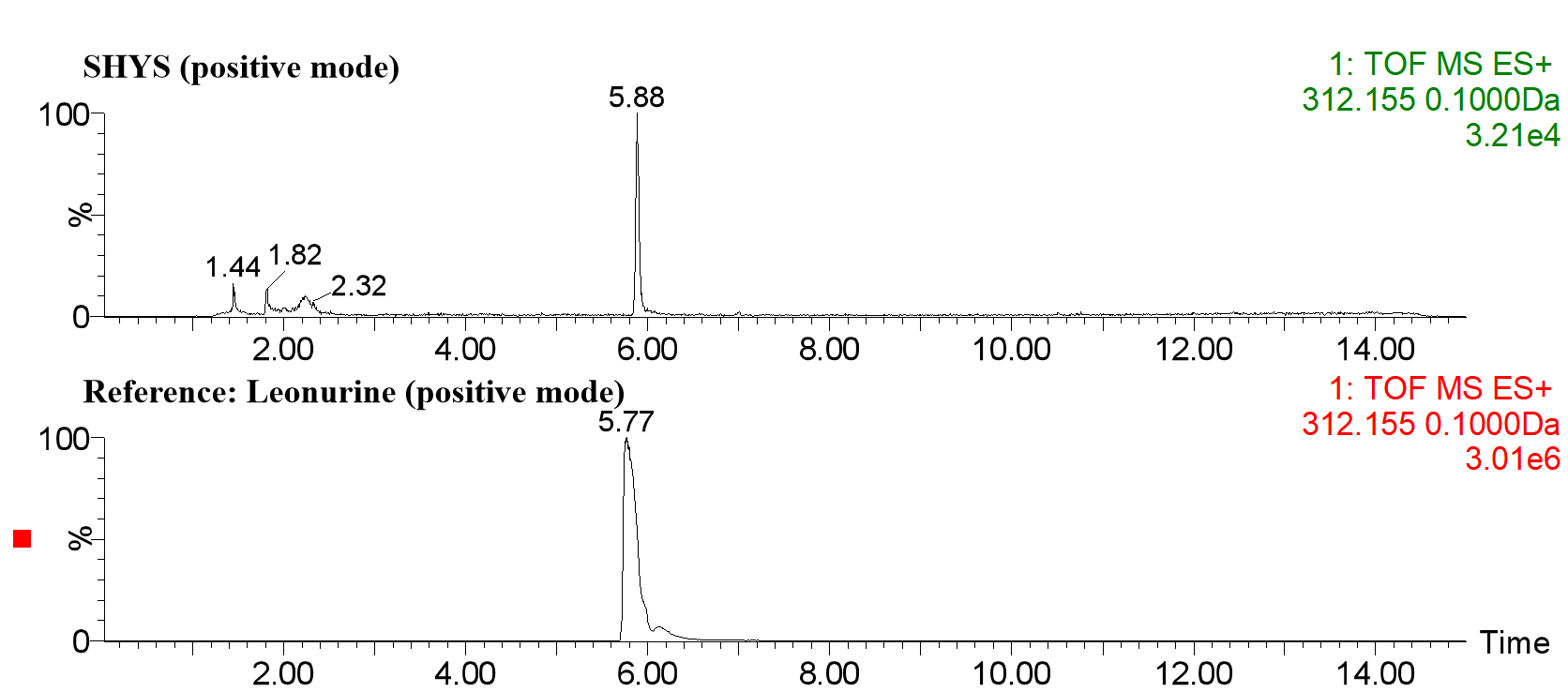
**

**t**

**
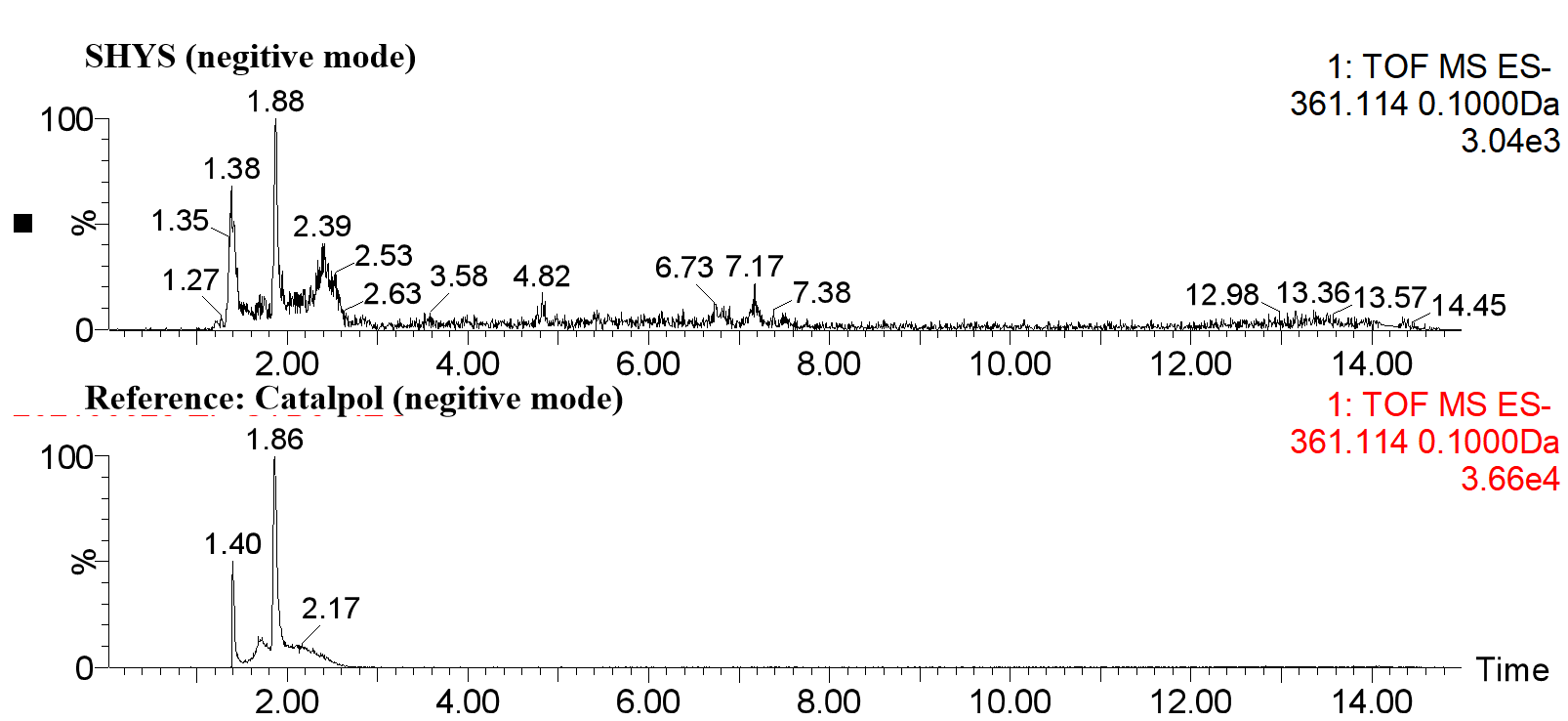
**

**Figure S4:** The chemical profiles of SHYS using UPLC-MS. (**a, b**) The total ion chromatogram in positive (**a**) and negative ion modes (**b**). (**c-t**) The main bioactive compounds of ferulic acid (**c**), atractylodin (d), tanshinone (e), astragaloside (f), hyperoside (g), loganin (h), gallic acid (i), morroniside (j), allantoin (k), oleanic acid (l), ginsenoside Rb1 (m), ginsenoside Re (n), ginsenoside Rg1 (o), paeoniflorin (p), vaccarin (q), pyrrolidinium (r), leonurine (s), and catalpol (t).

**TABLE S1 The characteristic fragment ions of reference standards in SHYS**

| **Marking**  **peak no.** | **Name** | **RT**  **(min)** | **Ion** |
| --- | --- | --- | --- |
| 1 | Ferulic Acid | 6.83 | [M-H]^-^ |
| 2 | Atractylodin | 5.49 | [M-H]^-^ |
| 3 | Tanshinone | 12.24 | [M+H]^+^ |
| 4 | Astragaloside | 9.49 | [M-H]^-^ |
| 5 | Hyperoside | 6.41 | [M-H]^-^ |
| 6 | Loganin | 5.49 | [M+H]^+^ |
| 7 | Gallic acid | 2.71 | [M-H]^-^ |
| 8 | Morroniside | 4.83 | [M-H]^-^ |
| 9 | Allantoin | 1.33 | [M-H]^-^ |
| 10 | Oleanic acid | 13.98 | [M-H]^-^ |
| 11 | Ginsenoside Rb1 | 8.61 | [M-H]^-^ |
| 12 | Ginsenoside Re | 7.25 | [M-H]^-^ |
| 13 | Ginsenoside Rg1 | 7.31 | [M+H]^+^ |
| 14 | Paeoniflorin | 6.05 | [M-H]^-^ |
| 15 | Vaccarin | 5.52 | [M-H]^-^ |
| 16 | Pyrrolidinium | 1.33 | [M+H]^+^ |
| 17 | Leonurine | 5.88 | [M+H]^+^ |
| 18 | Catalpol | 1.88 | [M-H]^-^ |
